# Supplementary material for: Medication adherence trajectories and association with risk factors and clinical outcomes in type 2 diabetes treatment
Source: PLoS One. 2026 Feb 20;21(2):e0342056. doi: 10.1371/journal.pone.0342056 (PMC12923057; doi:10.1371/journal.pone.0342056)

# Supporting information

**S1 Fig. Diagram highlighting study period and study design.** The figure illustrates the entire study period (1st January 2015 to 31 March 2021) including the recruitment period (1st January 2015 to 31 December 2019) and the individual one-year follow-up (FU) windows based on each patient’s index date (first OAD prescription, represented by a black square) and ended maximum on 31 December 2020. Clinical parameters evaluation was registered for each patient within 3 months before the index date (grey star) and within 3 months after the end of follow-up (grey hexagon). The adherence assessment within the follow-up window comprises the initiation (a), implementation (b), and discontinuation (c) phases. Each dashed line represents the sliding one-year follow-up window starting from the index date. The end of the study date was 31 March 2021.


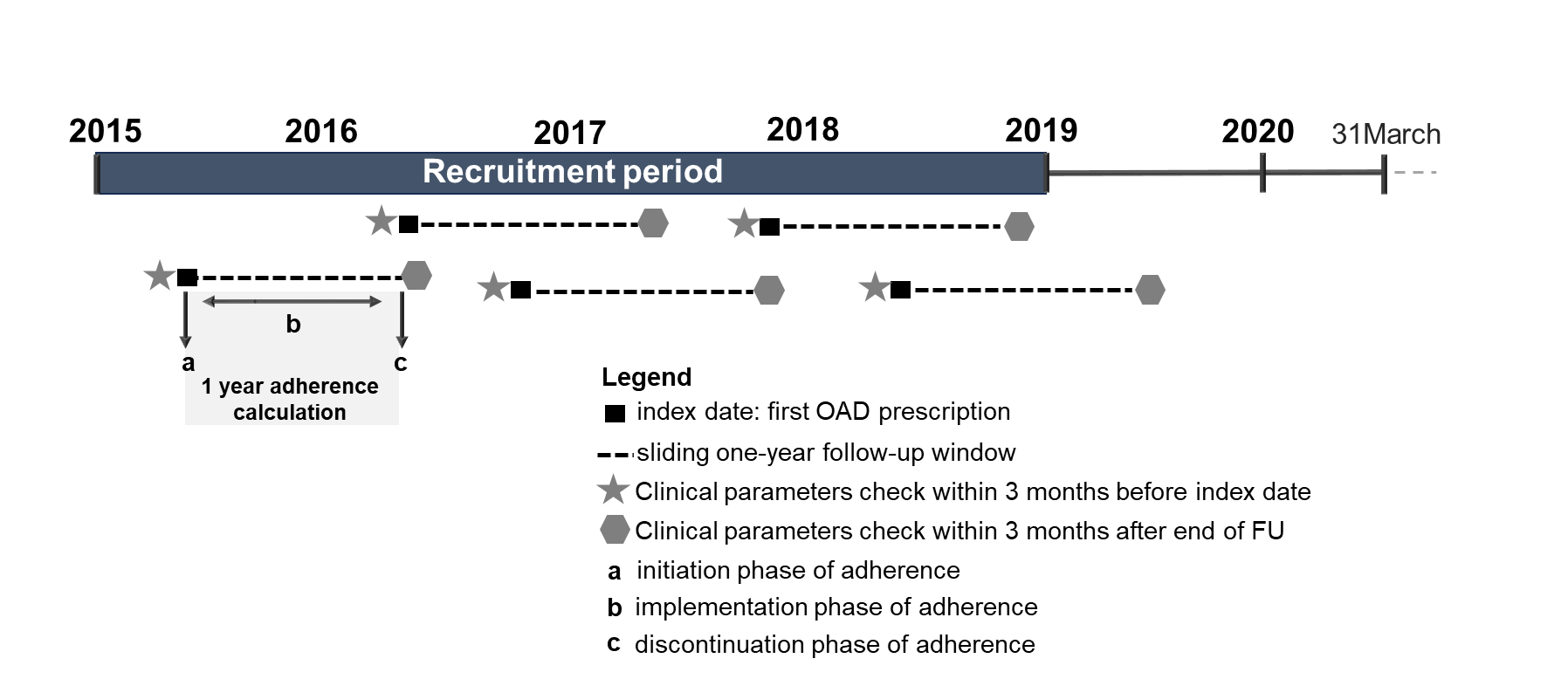

Supplement: S1 Fig — The figure illustrates the entire study period (1st January 2015–31 March 2021) including the recruitment period (1st January 2015–31 December 2019) and the individual one-year follow-up (FU) windows based on each patient’s index date (first OAD prescription, represented by a black square) and ended maximum on 31 December 2020. Clinical parameters evaluation was registered for each patient within 3 months before the index date (grey star) and within 3 months after the end of follow-up (grey hexagon). The adherence assessment within the follow-up window comprises the initiation (a), implementation (b), and discontinuation (c) phases. Each dashed line represents the sliding one-year follow-up window starting from the index date. The end of the study date was 31 March 2021. (DOCX) [file pone.0342056.s001.docx]
