# Supplementary figures and images for: Medication adherence trajectories and association with risk factors and clinical outcomes in type 2 diabetes treatment
Source: PLoS One. 2026 Feb 20;21(2):e0342056. doi: 10.1371/journal.pone.0342056 (PMC12923057; doi:10.1371/journal.pone.0342056)

# Supporting information

**S2 Fig. Study flow chart.**

**
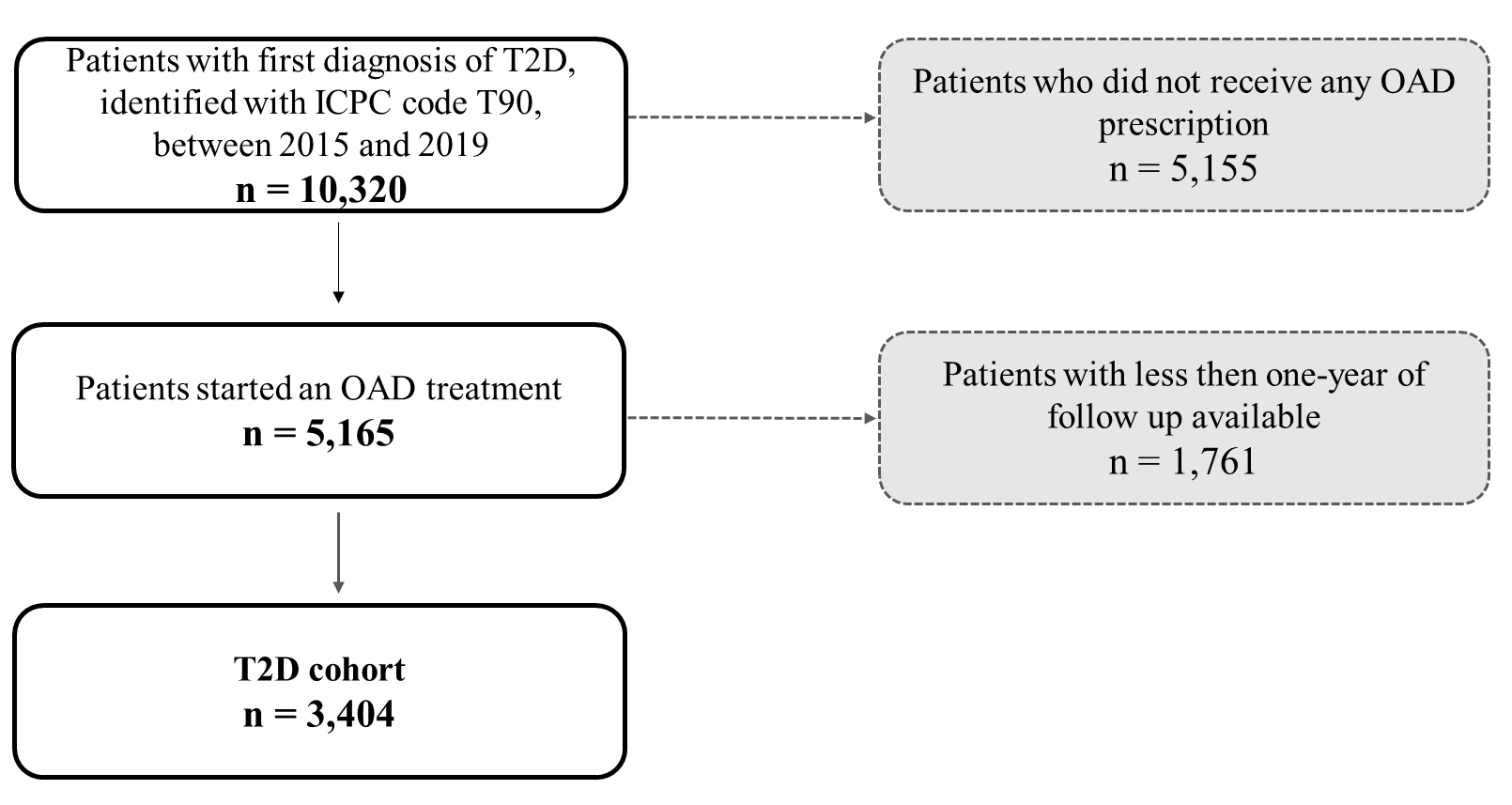
**

Supplement: S2 Fig — (DOCX) [file pone.0342056.s002.docx]
