# Supplementary material for: Medication adherence trajectories and association with risk factors and clinical outcomes in type 2 diabetes treatment
Source: PLoS One. 2026 Feb 20;21(2):e0342056. doi: 10.1371/journal.pone.0342056 (PMC12923057; doi:10.1371/journal.pone.0342056)

# Supporting information

**S3 Fig. Standardized cluster validity indices for selection of optimal number of adherence trajectories.** This figure illustrates the standardized values of these indices across different cluster solutions. The optimal number of adherence trajectories was determined by comparing model fit statistics across solutions ranging from two to six groups (k=2-6). To ensure robustness and consistency, multiple criteria were applied, including: i) Calinski-Harabasz index (CH – criterion 1; CH2 – criterion 2; CH3 – criterion 3); ii) Ray-Turi index (criterion 4); iii) Davies-Bouldin index (criterion 5). Each of these metrics evaluates cluster validity from a different perspective, such as compactness (intra-cluster similarity) and separation (inter-cluster dissimilarity). The Calinski-Harabasz indices (CH2, CH3) and Ray-Turi index reach their highest or near-highest standardized scores at four clusters, indicating a favorable balance between within-group cohesion and between-group separation. While CH and Davies-Bouldin indices slightly favor k=2 clusters, the overall multi-criteria profile supported k=4 clusters as the preferred solution. Robustness and stability of these k=4 clusters were further evaluated via bootstrap resampling, cluster-wise Jaccard similarities. Sensitivity analyses confirmed the selection [22–24].


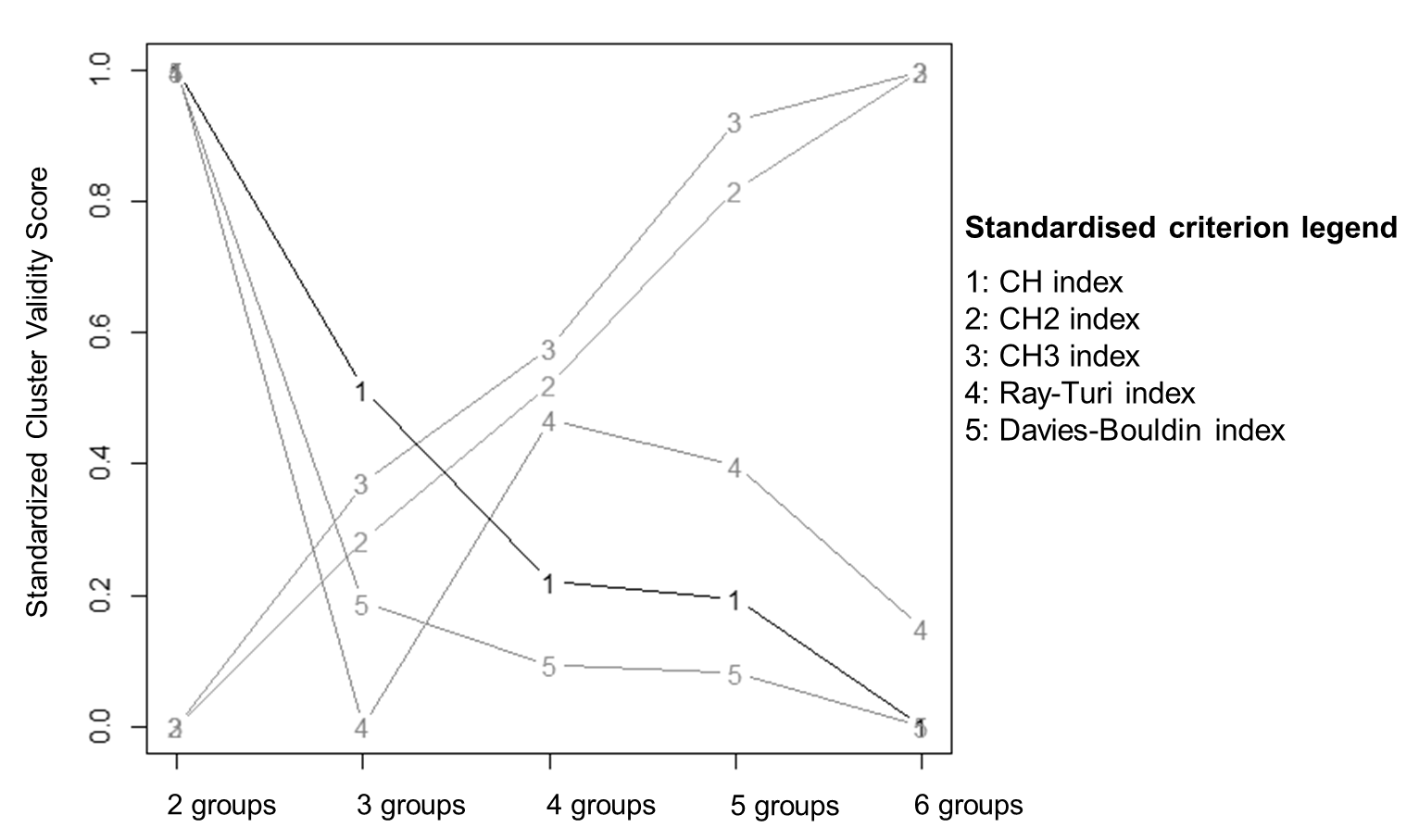

Supplement: S3 Fig — This figure illustrates the standardized values of these indices across different cluster solutions. The optimal number of adherence trajectories was determined by comparing model fit statistics across solutions ranging from two to six groups (k = 2–6). To ensure robustness and consistency, multiple criteria were applied, including: i) Calinski-Harabasz index (CH – criterion 1; CH2 – criterion 2; CH3 – criterion 3); ii) Ray-Turi index (criterion 4); iii) Davies-Bouldin index (criterion 5). Each of these metrics evaluates cluster validity from a different perspective, such as compactness (intra-cluster similarity) and separation (inter-cluster dissimilarity). The Calinski-Harabasz indices (CH2, CH3) and Ray-Turi index reach their highest or near-highest standardized scores at four clusters, indicating a favorable balance between within-group cohesion and between-group separation. While CH and Davies-Bouldin indices slightly favor k = 2 clusters, the overall multi-criteria profile supported k = 4 clusters as the preferred solution. Robustness and stability of these k = 4 clusters were further evaluated via bootstrap resampling, cluster-wise Jaccard similarities. Sensitivity analyses confirmed the selection [22–24]. (DOCX) [file pone.0342056.s003.docx]
