# Supplementary material for: Medication adherence trajectories and association with risk factors and clinical outcomes in type 2 diabetes treatment
Source: PLoS One. 2026 Feb 20;21(2):e0342056. doi: 10.1371/journal.pone.0342056 (PMC12923057; doi:10.1371/journal.pone.0342056)

# Supporting information

**S4 Fig. Longitudinal trajectories with a four adherence groups selection.** Each grey line represents the adherence trajectory. Each trajectory is the mean of monthly CMA9 over months 1–12 of a single T2D patient, grouped into one of the four adherence clusters (k=4). Areas with denser concentration of lines indicate greater similarity in patient behavior, which guided the clustering algorithm. The solid black line shows the mean CMA trajectory for each group, while the grey shaded area denotes the standard deviation, capturing the variability around the mean.


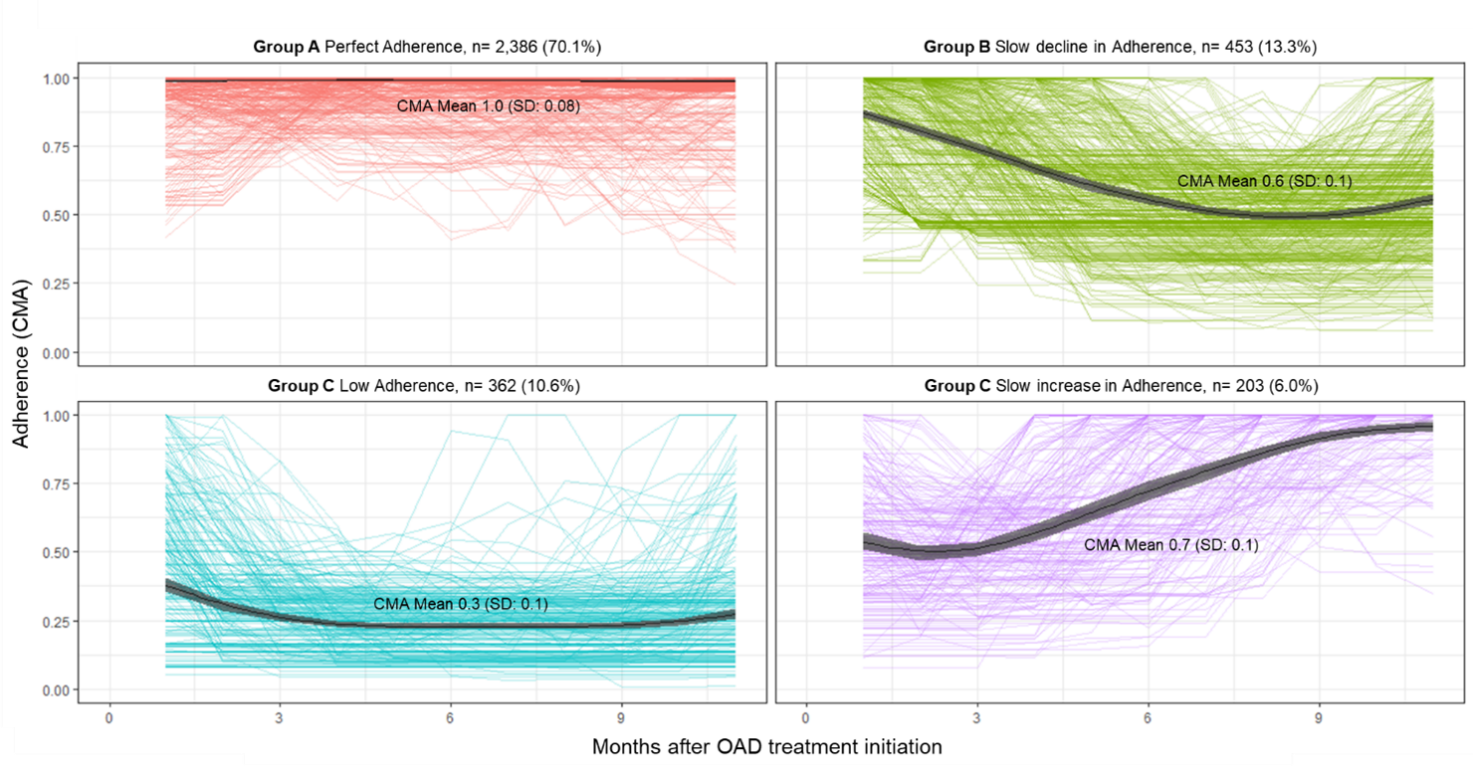

Supplement: S4 Fig — Each grey line represents the adherence trajectory. Each trajectory is the mean of monthly CMA9 over months 1–12 of a single T2D patient, grouped into one of the four adherence clusters (k = 4). Areas with denser concentration of lines indicate greater similarity in patient behavior, which guided the clustering algorithm. The solid black line shows the mean CMA trajectory for each group, while the grey shaded area denotes the standard deviation, capturing the variability around the mean. (DOCX) [file pone.0342056.s004.docx]
