# Supplementary material for: Medication adherence trajectories and association with risk factors and clinical outcomes in type 2 diabetes treatment
Source: PLoS One. 2026 Feb 20;21(2):e0342056. doi: 10.1371/journal.pone.0342056 (PMC12923057; doi:10.1371/journal.pone.0342056)
Supplement: S5 Fig — (DOCX) [file pone.0342056.s005.docx]

# Supporting information

**S5 Fig. Patients exceeding clinical outcomes T2D-related values stratified by adherence groups.**

**
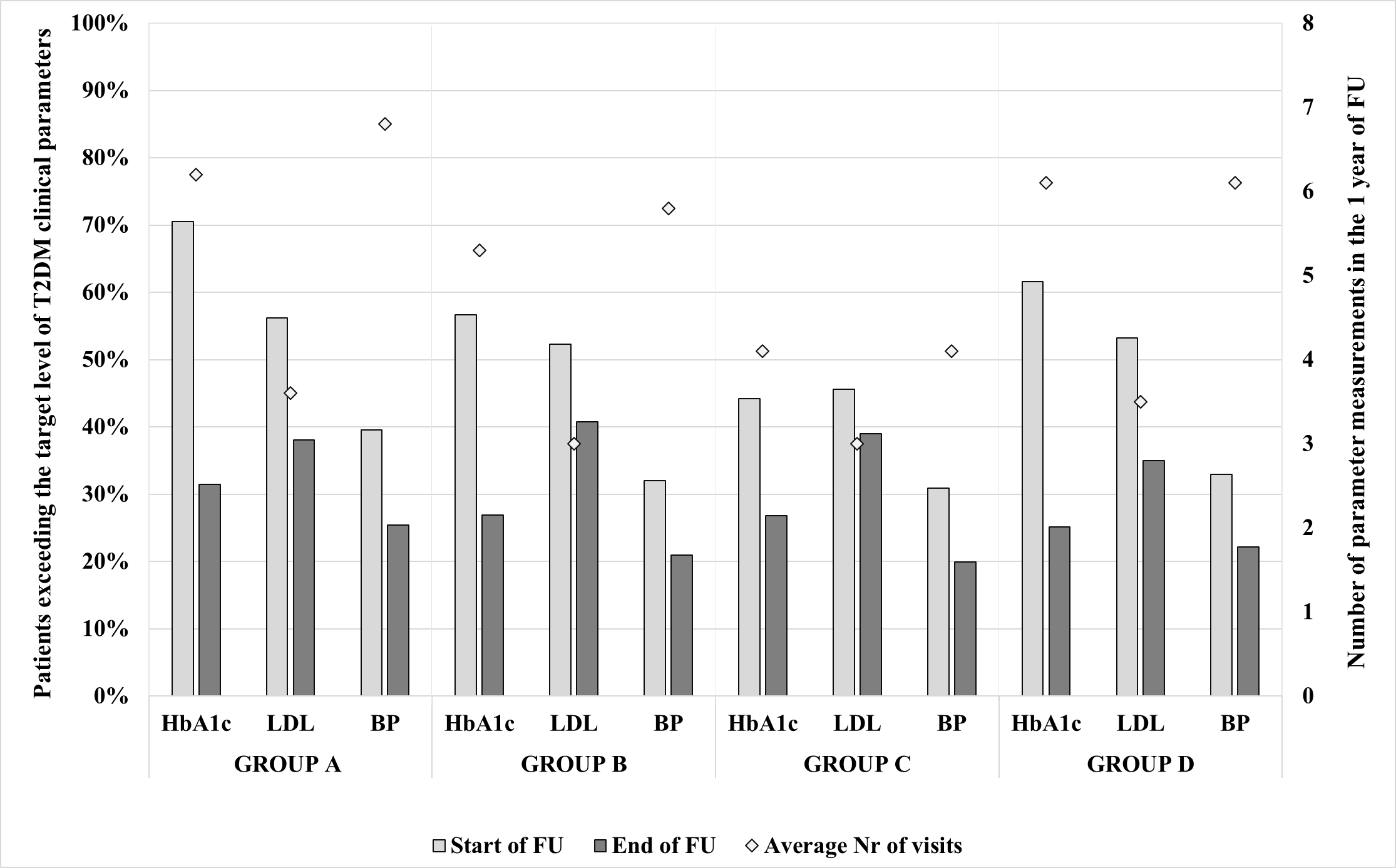
**
