# Supplementary material for: Medication adherence trajectories and association with risk factors and clinical outcomes in type 2 diabetes treatment
Source: PLoS One. 2026 Feb 20;21(2):e0342056. doi: 10.1371/journal.pone.0342056 (PMC12923057; doi:10.1371/journal.pone.0342056)

# Supporting information

**S7 Fig. Box-and-whisker plot correlating HbA1c levels at the start of follow-up and adherence groups.** Each box represents the interquartile range (IQR, 25th-75th percentile) of HbA1c values, with the horizontal line inside the box indicating the median HbA1c value. Whiskers extend to 1.5×IQR, and outliers are shown as individual points. The dashed horizontal line represents the clinical HbA1c target threshold, set at 53 mmol/mol (7%). The plot highlights differences in HbA1c distribution across adherence groups, with group A (perfect adherence) showing higher median values at the baseline compared to the other adherence groups.


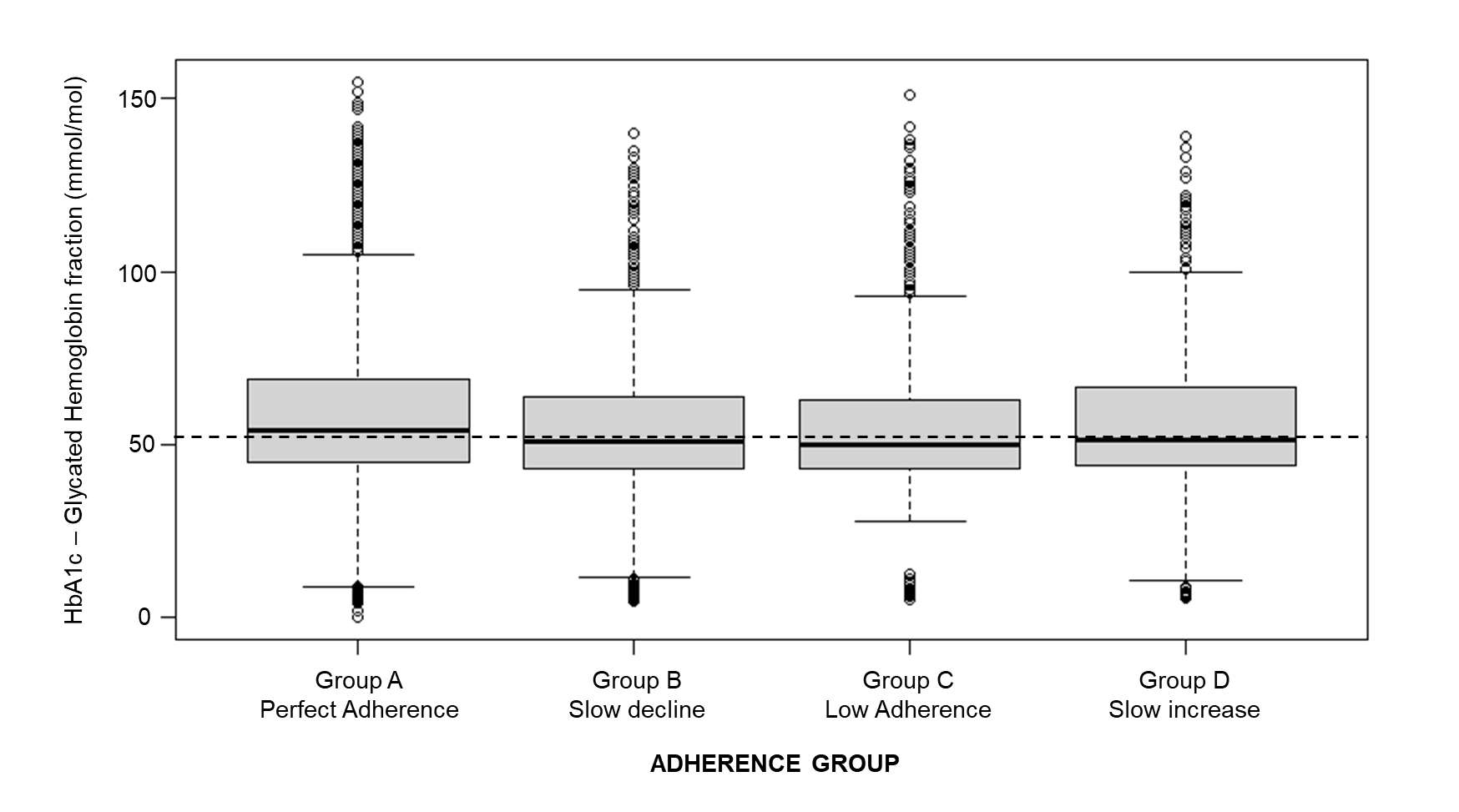

Supplement: S7 Fig — Each box represents the interquartile range (IQR, 25th-75th percentile) of HbA1c values, with the horizontal line inside the box indicating the median HbA1c value. Whiskers extend to 1.5 × IQR, and outliers are shown as individual points. The dashed horizontal line represents the clinical HbA1c target threshold, set at 53 mmol/mol (7%). The plot highlights differences in HbA1c distribution across adherence groups, with group A (perfect adherence) showing higher median values at the baseline compared to the other adherence groups. (DOCX) [file pone.0342056.s007.docx]
