# Supplementary material for: Medication adherence trajectories and association with risk factors and clinical outcomes in type 2 diabetes treatment
Source: PLoS One. 2026 Feb 20;21(2):e0342056. doi: 10.1371/journal.pone.0342056 (PMC12923057; doi:10.1371/journal.pone.0342056)
Supplement: S1 Table — (DOCX) [file pone.0342056.s008.docx]

# Supporting information

**S1 Table. Baseline patients’ characteristics.**

| **Characteristics** | **T2D cohort**  **N = 3,404** |
| --- | --- |
| **Males, *n (%)*** | 2,082 (61.2) |
| **Females, *n (%)*** | 1,322 (38.8) |
| **Age, *mean (SD)*** | 59.9 (13.3) |
| Aged under 25 years *(%)* | 19 (0.6) |
| Aged 26-39 years *(%)* | 179 (5.3) |
| Aged 40-59 years *(%)* | 1,499 (44.0) |
| Aged 60-79 years *(%)* | 1,439 (42.3) |
| Aged over 80 years *(%)* | 268 (7.9) |
| OAD treatment: **Biguanides*, n (%)*** | 3,149 (92.5) |
| OAD treatment: **Sulfonylureas*, n (%)*** | 237 (7.0) |
| OAD treatment: **Combinations of oral blood glucose lowering drugs*, n (%)*** | 5 (0.1) |
| OAD treatment: **Alpha glucosidase inhibitors*, n (%)*** | 1 (<0.1) |
| OAD treatment: **Thiazolidinediones*, n (%)*** | 2 (0.1) |
| OAD treatment: **DPP-4 inhibitors*, n (%)*** | 7 (0.2) |
| OAD treatment: **SGLT2 inhibitors*, n (%)*** | 1 (<0.1) |
| OAD treatment: **Other blood glucose lowering drugs, excl. insulins*, n (%)*** | 2 (0.1) |
| **High risk T2D patients, *n (%)*** | **396 (11.6)** |
| Angina pectoris | 64 (16.2) |
| Acute myocardial infarction | 105 (26.5) |
| Other and chronic ischaemic heart disease | 61 (15.4) |
| Heart failure | 62 (15.7) |
| Stroke/cerebrovascular accident | 69 (17.4) |
| Atherosclerosis | 16 (4.0) |
| Other arterial obstruction/pheriph. vascular disease | 33 (8.3) |
| Chronic alcohol abuse | 2 (0.5) |
| Obesity (BMI>30) | 13 (3.3) |
| Symptoms/complaints kidney | 5 (1.3) |
| **Charlson comorbidity score, average (SD)** | 2.0 (9.7) |
| CCI Low score (0-1), n (%) | 2,984 (87.7) |
| CCI Mild score (2-3), n (%) | 85 (2.5) |
| CCI Severe score (≥4), n (%) | 335 (9.8) |
| **Comorbid conditions, mean (SD)** | 2.4 (2.3) |
| **Comorbid conditions, median (IQR)** | 2 (1-3) |
| **Prescriptions per year, mean (SD)** | 34.5 (53.7) |
| **Prescriptions per year, median (IQR)** | 20 (12-33) |
| Concomitant medication: **Lipid Modifying Agents (C10), *n (%)*** | 1,827 (53.7) |
| Concomitant medication: **Agents acting on Renin-Angiotensin System (C09), *n (%)*** | 1,211 (35.6) |
| Concomitant medication: **Antithrombotic Agents (B01), *n (%)*** | 1,006 (29.6) |
| Concomitant medication: **Beta Blocking Agents (C07), *n (%)*** | 892 (26.2) |
| Concomitant medication: **Diuretics (C03), *n (%)*** | 808 (23.7) |
| Concomitant medication: **Psycholeptics (N05)** | 578 (17.0) |
| Concomitant medication: **Calcium Channel Blockers (C08), *n (%)*** | 545 (16.0) |
| Concomitant medication: **Drugs for Obstructive Airway Diseases (R03), *n (%)*** | 509 (15.0) |
| Concomitant medication: **Psychoanaleptics (N06), *n (%)*** | 457 (13.4) |
| Other chronic comordibity: **Hypertension, *n (%)*** | 750 (22.0) |
| Other chronic comordibity: **Lipid metabolism disorder, *n (%)*** | 270 (7.9) |
| Other chronic comordibity: **Asthma, *n (%)*** | 148 (4.3) |
| Other chronic comordibity: **COPD, *n (%)*** | 135 (4.0) |
| Other chronic comordibity: **Atrial fibrillation, *n (%)*** | 112 (3.3) |
| Other chronic comordibity: **Depressive disorder, *n (%)*** | 103 (3.0) |
| Other chronic comordibity: **Gout, *n (%)*** | 99 (2.9) |
| Other chronic comordibity: **Hypothyroidism/myxoedema, *n (%)*** | 95 (2.8) |
