# Supplementary material for: Medication adherence trajectories and association with risk factors and clinical outcomes in type 2 diabetes treatment
Source: PLoS One. 2026 Feb 20;21(2):e0342056. doi: 10.1371/journal.pone.0342056 (PMC12923057; doi:10.1371/journal.pone.0342056)
Supplement: S2 Table — (DOCX) [file pone.0342056.s009.docx]

# Supporting information

**S2 Table. Adherence phases and groups.**

| **Adherence Groups** | **T2D cohort** | **Group A**  **Perfect Adherence** | **Group B**  **Slow decline** | **Group C**  **Low Adherence** | **Group D**  **Slow increase** |
| --- | --- | --- | --- | --- | --- |
| **T2D Cohort, *n*** | **3,404** | **2,386** | **453** | **362** | **203** |
| **T2D Cohort, *%*** | **100%** | **70.1%** | **13.3%** | **10.6%** | **6.0%** |
| **Initiation**  Only 1 OAD prescription, *n (%)* | 30 (0.9) | - | - | 25 (6.9) | 5 (2.5) |
| **Implementation**  CMA, mean (SD) | 0.8 (0.3) | 1.0 (<0.1) | 0.6 (0.1) | 0.3 (0.1) | 0.7 (0.1) |
| **Discontinuation/Persistence**  1-year persistent patients, *n (%)* | 2,879 (84.6) | 2,358 (98.8) | 281 (62) | 94 (26) | 147 (72.4) |
