# Supplementary material for: Medication adherence trajectories and association with risk factors and clinical outcomes in type 2 diabetes treatment
Source: PLoS One. 2026 Feb 20;21(2):e0342056. doi: 10.1371/journal.pone.0342056 (PMC12923057; doi:10.1371/journal.pone.0342056)
Supplement: S3 Table — Insulin addition was defined as the first insulin prescription initiated after the index date (first OAD prescription) within the 12-month follow-up. Patients with any insulin before index were excluded from the overall T2D cohort. Percentages are within-group (the denominator is equal to the number of patients in each adherence group).Time at insulin addition is the number of days from index to the first post-index insulin prescription (mean value and standard deviation, SD). (DOCX) [file pone.0342056.s010.docx]

# Supporting information

**S3 Table. Post-index insulin initiation within the one-year follow-up, by adherence trajectory group.** Insulin addition was defined as the first insulin prescription initiated after the index date (first OAD prescription) within the 12-month follow-up. Patients with any insulin before index were excluded from the overall T2D cohort. Percentages are within-group (the denominator is equal to the number of patients in each adherence group).Time at insulin addition is the number of days from index to the first post-index insulin prescription (mean value and standard deviation, SD).

| **Adherence Groups** | **Group A Perfect Adherence** | **Group B Slow decline** | **Group C Low Adherence** | **Group D**  **Slow increase** |
| --- | --- | --- | --- | --- |
| **T2D Cohort**, *n (%)* | **2,386 (70.1)** | **453 (13.3)** | **362 (10.6)** | **203 (6.0)** |
| **Number of patients start insulin**, *n (%)* | 188 (7.9) | 55 (12.1) | 63 (17.4) | 21 (10.3) |
| **Time at insulin addition start**, *mean days (SD)* | 69 (91.0) | 48 (70.7) | 33 (78.0) | 94 (113.8) |
