# Supplementary material for: Medication adherence trajectories and association with risk factors and clinical outcomes in type 2 diabetes treatment
Source: PLoS One. 2026 Feb 20;21(2):e0342056. doi: 10.1371/journal.pone.0342056 (PMC12923057; doi:10.1371/journal.pone.0342056)
Supplement: S4 Table — (DOCX) [file pone.0342056.s011.docx]

# Supporting information

**S4 Table. Outcome Measures from the NHG Standaards for T2D monitoring.**

| **Clinical parameters** | **Check-ups** | **Outcome measure** | **Clinical target value** |
| --- | --- | --- | --- |
| **Measure glycated hemoglobin** | Quarterly | Hemoglobin A1c (HbA1c) level | ≤53 mmol/mol / ≤7% |
| **Measure LDL level** | Quarterly | Low-density lipoprotein (LDL) level | < 2.8 mmol/L |
| **Measure body weight** | Quarterly | Body Mass Index (BMI) | < 27 |
| **Measure blood pressure** | Quarterly | Blood Pressure (BP) | <140/90 mmHg |
