# Supplementary material for: Medication adherence trajectories and association with risk factors and clinical outcomes in type 2 diabetes treatment
Source: PLoS One. 2026 Feb 20;21(2):e0342056. doi: 10.1371/journal.pone.0342056 (PMC12923057; doi:10.1371/journal.pone.0342056)
Supplement: S5 Table — (DOCX) [file pone.0342056.s012.docx]

# Supporting information

**S5 Table. HbA1c (mmol/mol) levels by adherence trajectory: baseline vs end of one-year follow-up.**

| **Adherence Group** | **n patients** | **Mean HbA1c at baseline** | **Mean HbA1c at end of FU** | **Mean change**  **(end-baseline)** |
| --- | --- | --- | --- | --- |
| **A Perfect Adherence** | **2,386** | 75.93 | 53.27 | −22.66 |
| **B Slow decline in Adherence** | **453** | 70.12 | 52.64 | −17.48 |
| **C Low Adherence** | **362** | 66.25 | 56.09 | −10.15 |
| **D Slow increase in Adherence** | **203** | 71.57 | 51.51 | −20.06 |
