# Supplementary material for: Medication adherence trajectories and association with risk factors and clinical outcomes in type 2 diabetes treatment
Source: PLoS One. 2026 Feb 20;21(2):e0342056. doi: 10.1371/journal.pone.0342056 (PMC12923057; doi:10.1371/journal.pone.0342056)
Supplement: S6 Table — The outcome HbA1c level measured in mmol/mol. Fixed effects: adherence group with Group A (perfect adherence) as reference, months since index date, and their interactions. Random intercepts and slopes at the patient level. (DOCX) [file pone.0342056.s013.docx]

# Supporting information

**S6 Table. Linear mixed-effects model for HbA1c by adherence trajectory and time.** The outcome HbA1c level measured in mmol/mol. Fixed effects: adherence group with Group A (perfect adherence) as reference, months since index date, and their interactions. Random intercepts and slopes at the patient level.

| **Term** | **Estimate** | **95% CI (lower)** | **95% CI (upper)** | **p-value** |
| --- | --- | --- | --- | --- |
| **Intercept** | 6.84 | 6.73 | 6.95 | <0.001 |
| **Group B (vs A)** | −5.93 | −8.57 | −3.29 | 1.08×10 ^–5^ |
| **Group C (vs A)** | −6.78 | −10.05 | −3.52 | 4.67×10 ^–5^ |
| **Group D (vs A)** | −4.03 | −7.84 | −0.22 | 0.038 |
| **Months since index date** | −2.93 | −3.08 | −2.78 | <0.001 |
| **Months x Group B** | 0.44 | 0.08 | 0.80 | 0.016 |
| **Months x Group C** | 1.07 | 0.61 | 1.52 | 4.20×10 ^–6^ |
| **Months x Group D** | 0.45 | −0.09 | 0.99 | 0.104 |
