# Supplementary material for: Medication adherence trajectories and association with risk factors and clinical outcomes in type 2 diabetes treatment
Source: PLoS One. 2026 Feb 20;21(2):e0342056. doi: 10.1371/journal.pone.0342056 (PMC12923057; doi:10.1371/journal.pone.0342056)
Supplement: S7 Table — Slopes are derived from the linear mixed-effects model. Values represent the average monthly change in HbA1c (mmol/mol) over the one-year follow-up. The 12-month change is the monthly slope x 12. (DOCX) [file pone.0342056.s014.docx]

# Supporting information

**S7 Table. HbA1c slopes and association with adherence trajectories.** Slopes are derived from the linear mixed-effects model. Values represent the average monthly change in HbA1c (mmol/mol) over the one-year follow-up. The 12-month change is the monthly slope x 12.

| **Adherence group** | **Monthly slope**  **(mmol/mol per month)** | **Approx. 12-month change**  **(mmol/mol)** |
| --- | --- | --- |
| **A Perfect Adherence** | −2.93 | −35.16 |
| **B Slow decline in Adherence** | −2.49 | −29.88 |
| **C Low Adherence** | −1.86 | −22.36 |
| **D Slow increase in Adherence** | −2.48 | −29.79 |
