# Supplementary material for: Medication adherence trajectories and association with risk factors and clinical outcomes in type 2 diabetes treatment
Source: PLoS One. 2026 Feb 20;21(2):e0342056. doi: 10.1371/journal.pone.0342056 (PMC12923057; doi:10.1371/journal.pone.0342056)
Supplement: Attachment — Submitted filename: Response to Reviewers Letter.docx [file pone.0342056.s015.docx]

# **Response to Reviewer #1 Comments**

**Question 1: The entire manuscript needs revision for clarity and verbosity should be reduced throughout.**

**Answer 1:** Dear reviewer we accepted your main concern, so since April, we have been working intensively on improving the clarity and transparency of the manuscript, both in writing and in presenting the results through figures and textual descriptions. We have focused on improving linguistic accuracy. We have significantly shortened and simplified long and complex sentences, eliminated unclear and repetitive statements, and carefully revised the entire manuscript with language and content editing, where appropriate. During these months we also mainly focused on improving methodological transparency, including detailed labelling and description of each figure, both in the main text and in the supplementary material. Hence, the entire manuscript has been revised to improve overall readability. We believe that the revised version represents a significant improvement in both clarity and transparency, and we resubmitted it for journal consideration.

**Question 2: Add a section on limitations discussing confounding, data completeness, generalizability, and potential biases.**

**Answer 2:** In the revised version of the manuscript, we have included a dedicated section at the end of the discussion section, where we address key limitations. Specifically, we acknowledge that the study has several limitations that should be considered, primarily related to data completeness, potential biases, and the nature of prescription data. We used prescription records from the Nivel-PCD database to estimate adherence. However, these records cannot confirm whether patients collected or took their prescribed medication. To address this, we considered at least two prescriptions within the observation period as a proxy for treatment initiation and continuation. Additionally, clinical and demographic data were included only when essential fields were available. Records with missing clinical outcome measurements or incomplete patient information were excluded, and this may have introduced selection bias and could limit the generalizability of our findings. Lastly, the study cohort included only patients with a first recorded diagnosis of T2D, and we were unable to confirm whether they had been prescribed OADs prior to this diagnosis. Hence, a hypothetical prior exposure to an OAD treatment could have influenced adherence patterns that were not captured in this dataset. We addressed also reported all these aspects within this new version of the manuscript in lines 363–378.

**Question 3: Also, improve methodological transparency, especially around clustering decisions and variable selection.**

**Answer 3:** Dear reviewer, we mainly focused on this in this new revision. Hence, in respect to the clustering number decision, we have reinforced the methodology section and the supporting information file.

Indeed, in the supplementary file, we have added S3 Fig titled “Standardized cluster validity indices for selection of optimal number of adherence trajectories” (not present in the past version) with clarification regarding the indices used to plot and choose number of adherence groups. Particularly, the figure represents a visual representation of the standardized values of cluster validation methods used to determine the optimal number of cluster trajectories. A footnote below the S3 Fig was also added as follow: “*The optimal number of adherence trajectories was determined by comparing model fit statistics across solutions ranging from two to six groups. To ensure robustness and consistency, multiple criteria were applied, including: i) Calinski-Harabasz index (CH – criterion 1; CH2 – criterion 2; CH3 – criterion 3); ii) Ray-Turi index (criterion 4); iii) Davies-Bouldin index (criterion 5). Each of these metrics evaluates cluster validity from a different perspective, such as compactness (intra-cluster similarity) and separation (inter-cluster dissimilarity). This figure illustrates the standardized values of these indices across different cluster solutions. The Calinski-Harabasz indices (CH2, CH3) and Ray-Turi index reach their highest or near-highest standardized scores at four clusters, indicating a favorable balance between within-group cohesion and between-group separation. While the Calinski-Harabasz -CH and Davies-Bouldin indices slightly favor a smaller number of clusters (e.g., k=2). Given this multi-criteria convergence and the importance of clinical interpretability, the four-cluster solution was selected as the most appropriate*”. A brief specification was also given in the main text and can be now found in the in lines 171-178.

**Question 4: There is limited discussion on potential unmeasured confounders that may influence both adherence and outcomes (e.g., socioeconomic status, education, health literacy).**

**Answer 4:** As previously mentioned, we acknowledge that certain important variables, such as socioeconomic status, education level, and health literacy, were not available in our dataset and could not be accounted for in our study. In the revised version of the manuscript, we expanded the discussion section to explicitly state the possibility of cofounding due to unmeasured factors. These aspects are now addressed in the discussion section in lines 318-321.

**Question 5: Ensure consistency in citation format and update with more recent studies post-2021.**

**Answer 5:** We acknowledge that this inconsistency occurred. However, as part of the major revision process, we conducted a thorough literature review and have now updated the reference list to include more recent and relevant studies published since 2021 to date. These updated references have also been incorporated into the discussion section to better contextualize our findings within the current state of the field. Also, citation format has been revised according to the guidelines of the journal.

# **Response to Reviewer #2 Comments**

**Question 1: Thank you for giving me the opportunity of reviewing this paper about adherence trajectories in type 2DM and their relation with clinical parameters. Although I considered the topic interesting I have several concerns (mainly in methods) that I would suggest to address. They can find below:**

**Answer 1:** Dear reviewer we appreciated your detailed questions/comments. We have been working intensively on improving the clarity and transparency of the manuscript. During these months we also mainly focused on improving methodological transparency, including detailed labelling and description of each figure, both in the main text and in the supplementary material. Hence, the entire manuscript has been revised to improve overall readability. We believe that the revised version represents a significant improvement in both clarity and transparency, and we resubmitted it for journal consideration. We also provided to address your point-by-point revision both within the manuscript and here below within our answers.

**Question 2: Introduction. Authors refer to single (adherence) estimates in the introduction section. Could they give some example of this single estimates?**

**Answer 2:** We maily referred to the widespread use of summary measures such as the Proportion of Days Covered (PDC) or the Medication Possession Ratio (MPR). These metrics typically provide a single percentage value representing the proportion of time a patient is assumed to be adherent over a given period, usually compared to a binary threshold or cut-off point (e.g., 80%, as literature so far suggest). These metrics are commonly used in adherence research to classify patients as “adherent” or “non-adherent” at a single time point or over an entire observation window, without capturing how adherence may change dynamically over time. On the other hand, a longitudinal approach evaluating adherence across the three distinct phases of the process (according to the EMERGE guidelines: initiation, implementation, and discontinuation/persistence), offering a more detailed understanding of medication-taking behaviour over time.

We have now implemented and clarified this explanation in the introduction and the discussion sections of the revised manuscript.

**Question 3: Methods. Did all patients have the some follow-up period? Based on Supplementary figure I presume it is but it should be clearer explained in the main text. If it is like this, it would be clearer if authors specify that the observation period was 1 year or upon treatment discontinuation.**

**Answer 3:** The methods section has now been completely revised by including many more specifics related to the study design. Specifically, to be more transparent, we have also replaced the S1 Figure in the supporting information file “Diagram highlighting study period and the evaluation of three phases of medication adherence”. This figure now shows the study design in detail with a clear specification describing it in the footnote.

Specifically, the entire study period was from 1st January 2015 to 31 March 2021. This includes the recruitment period (1st January 2015 to 31 December 2019) and the individual one-year follow-up (FU) windows based on each patient’s index date (first OAD prescription) and ended maximum on 31 December 2020. Clinical parameters evaluation was registered for each patient within 3 months before the index date and within 3 months after the end of FU. Clinical parameters were also recorded during the entire one-year follow-up for each patient to address changes during the FU period. The overall study end date was on 31 March 2021.

**Question 4: What data did authors use to estimate adherence within prescription data? Prescription dates? How did authors assessed (operationalized) discontinuation or implementation?**

**Answer 4:** Medication adherence was computed according to the EMERGE guidelines, distinguishing the different stages of the adherence process as follows: i) initiation, patient who start the prescriber treatment; ii) implementation, the extent to which a patient’s actual OAD dosing corresponds to the prescribed dosing regimen, from initiation (index date) until the last dose; ii) discontinuation/persistence, i.e. the moment the patient stops taking the prescribed index OAD medication. This latter includes also the concept of, which was computed as the length of time between initiation (first OAD prescription at index date) and the last dose, which immediately precedes discontinuation. Initiation was calculated as having at least two prescriptions of any oral antidiabetic drug (OAD) within the one-year follow-up period. Patients with only one OAD prescription were flagged as “spot patients” or “non-initiators” and excluded from persistence calculation. Indeed, persistence calculation is time-to-event, for this reason it is not possible to determine whether the only one OAD prescription was actually taken by the patient or not (e.g., the patient does not start the prescribed treatment). Discontinuation/persistence phase was assessed by evaluating the duration of medication coverage for each prescription. A patient was considered non-persistent (discontinuer) if no subsequent prescription was recorded, or if the time to the next prescription exceeded twice the number of days covered by the previous one. This approach identifies gaps in treatment that indicate discontinuation during the follow-up period. Trajectory-based modelling, based on clustering approach, was used to calculate and plot implementation phase. Clustering method is a set of multivariate data analysis techniques aimed at selecting and grouping homogeneous items in a data set by using non-parametric statistics.

To improve clarity, this information has been fully detailed in the methods section of the revised manuscript, lines 96-115.

**Question 5: What was the adherence measurement used?**

**Answer 5:** The indicator used for the adherence calculation was the continuous multiple interval measure of medication availability/gaps (CMA) version 9. For the whole observation period, the CMA9 was computed as the ratio of theoretical medication use days to the adherence assessment period duration, accounting for prescriptions carryover and excluding end-period supply. The CMA9 established a consistent average adherence value by weighing days according to individual prescriptions ratios, calculated without overlap in one-month sliding windows, matching the typical delivery period between successive prescriptions. CMA is a time-sensitive estimate of medication availability across successive dispensing intervals. For this reason, CMA, comparing to other adherence indicators, particularly suited for trajectory-based modelling of adherence behaviours.

These aspects are now addressed within the manuscript in lines 153-159.

**Question 6: I do not fully understand why authors exclude patient who only received one prescription when prescription could easily have a duration of one year.**

**Answer 6:** We excluded patients with only one prescription during the follow-up period because, in the Dutch healthcare system where this study was conducted, oral antidiabetic drugs (OADs) are not prescribed with a coverage of one year. According to national prescribing practices in the Netherlands, the maximum duration of a prescription for chronic medications such as OADs is generally 90 days. This limit ensures appropriate clinical monitoring and is in line with guidelines issued by Dutch primary care standards (e.g., NHG-Standaarden).

As a result, a single prescription is unlikely to cover the full 12-month follow-up period. Including these patients in the adherence analysis would risk overestimating adherence and persistence, since we would not be able to determine whether the treatment was continued or discontinued after the initial prescription. To maintain methodological rigour and avoid introducing bias, we only included patients with at least two prescriptions in the follow-up period. This approach ensures that we can more reliably assess implementation and persistence over time.

We better explained this in lines 159-169 of the new manuscript version.

**Question 7: Did authors used trajectory analysis for estimate clusters of subjects with similar adherence behavior or with common characteristics (common risk factors, eg. Gender, age, comorbidities…)?**

**Answer 7:** In this study, trajectory analysis was used exclusively to identify clusters of patients with similar longitudinal adherence behaviors, not based on shared baseline characteristics such as age, gender, or comorbidities. The group-based trajectory approach was applied to daily adherence data, specifically, the continuous multiple-interval measure of medication availability (CMA), over a 12-month follow-up period. The aim of this clustering was to capture patterns of medication use over time, allowing us to distinguish dynamic adherence profiles (e.g., perfect adherence, slow decline, slow increase, and low adherence).

We better explained this in lines 112-188.

**Question 8: Some information about the % of patients with each clinical outcome recorded should be given.**

**Answer 8:** We have completely revised the results of the manuscript. You will now find more descriptions of the clinical outocomes in the results sub-section “Adherence groups and clinical outcomes”.

**Question 9: How were measured level of multimorbidity and polypharmacy?**

**Answer 9:** Multimorbidity was assessed using diagnosis data recorded in the Nivel Primary Care Database (Nivel-PCD). Specifically, we identified chronic conditions based on ICPC codes (International Classification of Primary Care) recorded by general practitioners. In line with widely accepted definitions, patients with two or more chronic conditions at baseline were classified as multimorbid.

Polypharmacy was defined as the concurrent use of five or more different medications at baseline.

Both definitions are now clearly reported in the revised version of the manuscript in lines 122-123.

**Question 10: What was the time unit in trajectory analysis?**

**Answer 10:** Although adherence (CMA) was calculated using daily-level data from prescription dates and days of supply, the trajectory analysis was performed using a monthly time unit. Specifically, the 12-month follow-up period was divided into 12 monthly intervals, and a monthly CMA was computed for each patient over these intervals. These monthly values were then used to identify longitudinal adherence trajectories.

Please refer to lines 157-159.

**Question 11: It is slightly confusing to me when authors say that they use refill data for estimating adherence but previously they had said they use prescription data. Refill data is dispensing data. Additionally, a bit of context should be added of how works pharmacy system in the Netherlands. What is the regular duration of prescriptions? If those prescription should be filled in pharmacies, how often should patients go to pharmacies to fill their prescriptions? Monthly? What information about refilling did authors use? Pills refilled? Refilling date? Are authors really considering prescription information?**

**Answer 11:** We confirm that we used prescription data from Nivel-PCD, which contains records of medications prescribed by general practitioners in the Netherlands. These records indicate that a medication was prescribed, including the prescription date and the number of days of supply intended, but do not provide information on whether the medication was dispensed or collected at the pharmacy. We better specified this and added a specification in the limitation section as follows: *“(..) We used prescription records from the Nivel-PCD database to estimate adherence. However, these records cannot confirm whether patients collected or took their prescribed medication. To address this, we considered at least two prescriptions within the observation period as a proxy for treatment initiation and continuation”.*

Therefore, we did not use refill or dispensing data. The terminology has been revised where necessary in the manuscript to consistently refer to prescription data, avoiding any confusion with refill data.

Additionally, in the Netherlands, prescriptions for oral antidiabetic medications (OADs) are usually issued for standardised durations, which vary slightly depending on the drug. In our study, we estimated the treatment coverage of each prescription based on Dutch pharmacological guidelines, which define expected durations per OAD class. To define discontinuation and persistence, we considered the time between consecutive prescriptions. If no new prescription was recorded within twice the coverage period of the previous medication (eg 180 days for OAD with 90-day coverage), the patient was classified as non-persistent.

These aspects are described in the revised methods section (pages 7-8).

**Question 12: What happens if a prescription duration is shorter than one year, if a doctor increases the prescribed dose or switches one OAD for another?**

**Answer 12:** In our methodology, if a patient received only one prescription for the index OAD (i.e., the first prescribed oral antidiabetic drug), and no further prescriptions were observed within twice the expected coverage duration (e.g., 180 days for a drug with a 90-day coverage), the patient was classified as non-persistent. Moreover, if the physician increased the dose of the same OAD, and a second prescription was issued before the calculated end of the first coverage period, the new prescription extended the overall coverage accordingly. While, if a switch occurred from the index OAD to another OAD, we considered this as a discontinuation of the index drug, and the patient was flagged as non-persistent to the initial treatment.

**Question 13: Regarding the analysis performed to estimate the change in HbA1c, did authors take into account time? What happens if one patient have several HbA1c measurements after or before the index prescription? Was the time of the measurement of HbA1c considered? It is not the same one measurement of HbA1c one month than 8 months after the index date.**

**Answer 13:** We understand your concern as in the previous version of the manuscript we were not comprehensive in our explanation of the measurements of clinical parameters. We have in this version completely revised the sub-section of the methods “clinical outcomes” (pages 6-7) improving clarity in the methodology.

More specifications have been added as follows: “*(..)* *Hence, clinical parameters were observed for each T2D patient within the 3 months before the index date and within the 3 months after the one-year follow-up period. Thus, the end of the entire study period was on 31 March 2021. If the T2D patient had several clinical parameter measurements before the index prescription or after the end of follow-up, the measurement closest in time to these two set dates was considered. Additionally, number of clinical measurements collected for each T2D patient within the one-year FU period were also retrieved to assess temporal variation and compare trends across adherence groups*”.

You can also refer to the now added S1 Figure in the supporting information which graphically explain this concept.

**Question 14: Results: In the methods section authors said that they exclude those patients who only had one prescription however this exclusion criteria is not reflected in the flow chart.**

**Answer 14:** We understand this question, hence we have improved the clarity of this statement, now correctly stated in the manuscript, i.e.: *“(..) Initiation was calculated as having at least two prescriptions of any oral antidiabetic drug (OAD) within the one-year follow-up period (excluding fo. Patients with only one OAD prescription were flagged as “spot patients” or “non-initiators” and excluded from persistence calculation. Indeed, persistence calculation is time-to-event, for this reason it is not possible to determine whether the only one OAD prescription was actually taken by the patient or not (e.g., the patient does not start the prescribed treatment)”.*

**Question 15: Data about insulin addition in each adherence group is very interesting, however the time at insuline addition shows a low relation with the effect (or non effect) of the medication. This effect is mainly valued based on Hb1Ac which is a marker that reflect the diabetes control in the last approximately 3 months… however the mean time at insulin addition is quite low to reflect the impact of the OAD. Some discussion about it would be acknowledged.**

**Answer 15:** We thank for this observation. We have indeed added this consideration within the limits of our study as follows: *“(..) Moreover, although insulin addition was evaluated as a potential inefficacy of the OAD treatment, the mean time to insulin addition was relatively short in our cohort. Therefore, the decision to initiate insulin treatment may not fully reflect the glycaemic effect of OAD therapy, especially considering that HbA1c reflects glycaemic control over the preceding 2–3 months.”*

**Question 15: In table 1, one of the charactertists in each group is number of prescriptions, however it seems a very high number of prescriptions having 39 or 20. What is the period considered to have these numbers?**

**Answer 15:** As now specified in the revised Table 1, the reported numbers reflect the total number of prescriptions per patient within the one-year follow-up period. These include all medications prescribed, not only the index OAD.

These values can be supported by the typical prescription patterns for patients with T2D, who often receive drugs for comorbid conditions such as hypertension, hyperlipidaemia, or cardiovascular disease. For example, an OAD with a daily dosing regimen and a standard coverage of about 30 days may require around 12 prescriptions per year. When combined with treatments for other chronic conditions, the total number of prescriptions can reach 20 to 40 per year, indicating the complex polypharmacy regimens frequently observed in these patients.

**Question 16: How did authors deal with those patients who had missing data in the clinical parameters?**

**Answer 16:** Records with missing clinical outcome measurements or incomplete patient information were excluded, and this may have introduced selection bias and could limit the generalizability of our findings. We have now specified this in the limits of the study in the new version of the manuscript at the end of the discussions (lines 369-371).

**Question 17: It seems that the adherence group with worse clinical parameters were Group A, perfect adherence, some discussion about it would be acknowledged.**

**Answer 17:** We agree with this observation. We have implemented the manuscript discussing more this finding. Please refer to lines 291-306 of this revised discussion section.

**Question 18: In Figure 1 it seems that in all adherence group there were a number of patients who showed values around 0, it seems strange as it not feasible having a value of 0. Additionally, I would recommend a figure with a more clear definition.**

**Answer 18:** We appreciate this pertinent comment as, indeed, the values around 0 for HbA1c were due to erroneously recorded or incomplete laboratory results in the raw data. We carefully re-checked the dataset and re-ran the analysis, which allowed us to regenerate Figure 1. The updated version of Figure 1 has now been included in the revised manuscript.

**Question 18: Discussion: In contrast with other papers in the field, results showed that older patients are less adherent. I would acknowledge some comparison with existing literature to discuss this result. Similarly with data of 70% patients in the perfect adherence group.**

**Answer 18:** We have incorporated all these aspects into the discussion now in the new manuscript version. The comments on the correlation between adherence and age groups are on lines 307-321 while those on the 70% patients in the perfect adherence group are discussed on lines 322-333.
