# Supplementary material for: Medication adherence trajectories and association with risk factors and clinical outcomes in type 2 diabetes treatment
Source: PLoS One. 2026 Feb 20;21(2):e0342056. doi: 10.1371/journal.pone.0342056 (PMC12923057; doi:10.1371/journal.pone.0342056)
Supplement: Attachment — Submitted filename: Response to Reviewers.docx [file pone.0342056.s016.docx]

**Response to Reviewers**

**PONE-D-25-41866**

**Medication adherence trajectories to predict risk factors and clinical outcomes in type 2 diabetes treatment**

**Academic Editor Comments**

**Dear Dr. Mucherino, thank you for submitting your manuscript to PLOS ONE. After careful consideration, we feel that it has merit but does not fully meet PLOS ONE’s publication criteria as it currently stands. Therefore, we invite you to submit a revised version of the manuscript that addresses the points raised during the review process.**

**Response:** Dear Academic Editor, we appreciate your consideration and the valuable input from the three reviewers. We have addressed all reviewer comments and prepared a substantially revised version of the manuscript and supplementary material. Below in this report you’ll find all the detailed responses to reviewers also addressed in the manuscript.

**Journal Requirements**

**Comment 1. Please ensure that your manuscript meets PLOS ONE's style requirements, including those for file naming. The PLOS ONE style templates can be found at**

[**https://journals.plos.org/plosone/s/file?id=wjVg/PLOSOne_formatting_sample_main_body.pdf**](https://journals.plos.org/plosone/s/file?id=wjVg/PLOSOne_formatting_sample_main_body.pdf) **and**

[**https://journals.plos.org/plosone/s/file?id=ba62/PLOSOne_formatting_sample_title_authors_affiliations.pdf**](https://journals.plos.org/plosone/s/file?id=ba62/PLOSOne_formatting_sample_title_authors_affiliations.pdf)**.**

**Response:** We have revised the submission to comply with PLOS ONE style and file-naming requirements.

**Comment 2. We note that you have indicated that there are restrictions to data sharing for this study. PLOS only allows data to be available upon request if there are legal or ethical restrictions on sharing data publicly.**

**For more information on unacceptable data access restrictions, please see http://journals.plos.org/plosone/s/data-availability#loc-unacceptable-data-access-restrictions.**

**Before we proceed with your manuscript, please address the following prompts:**

**a) If there are ethical or legal restrictions on sharing a de-identified data set, please explain them in detail (e.g., data contain potentially identifying or sensitive patient information, data are owned by a third-party organization, etc.) and who has imposed them (e.g., a Research Ethics Committee or Institutional Review Board, etc.). Please also provide contact information for a data access committee, ethics committee, or other institutional body to which data requests may be sent.**

**b) If there are no restrictions, please upload the minimal anonymized data set necessary to replicate your study findings to a stable, public repository and provide us with the relevant URLs, DOIs, or accession numbers. For a list of recommended repositories, please see**

**https://journals.plos.org/plosone/s/recommended-repositories. You also have the option of uploading the data as Supporting Information files, but we would recommend depositing data directly to a data repository if possible.**

**We will update your Data Availability statement on your behalf to reflect the information you provide.**

**Response:** Data of the Nivel Primary Care Database (Nivel-PCD) contain potentially identifying or sensitive patient information. Access to data in Nivel-PCD is subject to Nivel-PCD governance codes. Requests for access to the data can be directed at [datarequest@nivel.nl](mailto:datarequest@nivel.nl). Restrictions include approval by the appropriate Nivel-PCD governance bodies (privacy committee and steering committee).

**Comment 3. In the online submission form you indicate that your data is not available for proprietary reasons and have provided a contact point for accessing this data. Please note that your current contact point is a co-author on this manuscript. According to our Data Policy, the contact point must not be an author on the manuscript and must be an institutional contact, ideally not an individual. Please revise your data statement to a non-author institutional point of contact, such as a data access or ethics committee, and send this to us via return email. Please also include contact information for the third party organization, and please include the full citation of where the data can be found.**

**Response:** Data of the Nivel Primary Care Database (Nivel-PCD) contain potentially identifying or sensitive patient information. Access to data in Nivel-PCD is subject to Nivel-PCD governance codes. Requests for access to the data can be directed at [datarequest@nivel.nl](mailto:datarequest@nivel.nl). Restrictions include approval by the appropriate Nivel-PCD governance bodies (privacy committee and steering committee).

**Comment 4. If the reviewer comments include a recommendation to cite specific previously published works, please review and evaluate these publications to determine whether they are relevant and should be cited. There is no requirement to cite these works unless the editor has indicated otherwise.** 
**Response:** Thanks for the notice. No citations were recommended by the reviewers.

**Reviewers’ Comments**

**Reviewer #1 Comments**

**Comment 1. The data and the topic of the manuscript is interesting. However, the manuscript itself is difficult to read because of long paragraphs and complex sentences. The authors should condense sentences throughout the text. Abstract should be clearly structured.
Response:** Dear Reviewer, many thanks for the valuable comments provided. We understand this observation. We have revised the whole manuscript for clarity, shortening long sentences, splitting dense paragraphs, and removing redundant phrasing. We also reformatted the Abstract into a fully structured format (Background, Methods, Results, Conclusions).

**Comment 2. The definition of follow-up is not clearly explained in methods section. Figure 1 clarifies this, but some of the information of figure 1 should be explained also in the text.
Response:** We have now made the follow-up definition explicit also in the methods section. We now aligned the text with the scheme shown in Figure 1.

**Comment 3. Does baseline characteristics refer to index date (table 1)?
Response:** Yes. Baseline characteristics are assessed at the index date, defined as the date of the first OAD prescription. We better specified this in the new revised version of the manuscript.

**Comment 4. Because the adherence is defined based on the prescription of medication, the system of prescription data should be clarified. For how long period is one prescription? Is there any system to check whether patient has purchased the medication prescribed?
Response:** We fully understand this important clarification request. In this study (based on Nivel-PCD database) we observed general-practice prescribing records (prescription date, drug/ATC code, quantity/instructions when available, diagnosis, and so on). We do not have pharmacy dispensing claims. This is of course a study limitation that should be considered, primarily related to potential biases, related, of course, to the nature of prescription data itself. To address this, we considered at least two prescriptions within the observation period as a proxy for treatment initiation and continuation but, we cannot directly verify whether a patient really collected or ingested the medication. This aspect is highlighted in the limits’ declaration in the discussion section.

Moreover, the duration of all OAD prescriptions is not fixed, as each OAD have a different coverage period. Hence, to standardize exposure, we derived days’ supply from the recommended daily dosing for each OAD class using national sources already cited in the manuscript (NHG guideline and the Medicines Information Bank of the Dutch MEB). For example, sulfonylureas, thiazolidinediones, DPP-4 inhibitors, and SGLT2 inhibitors are typically once daily; combinations/α-glucosidase inhibitors 1-3 times/day; biguanides 2-3 times/day (extended-release once daily). We used these dosing rules to compute expected coverage per prescription and to construct monthly CMA9 trajectories. This is reported in the manuscript within the Statistical Analyses section.

Additionally, because dispensing is unavailable, persistence phase was computerised from prescribing patterns. A patient was classified non-persistent when no subsequent prescription was observed or when the interval to the next prescription exceeded 2x the expected coverage of the previous one. Patients with only one OAD prescription during follow-up were reported under initiation but excluded from persistence estimation to avoid misclassification (eg, primary non-initiation versus very long coverage cannot be distinguished with a single prescription).We’ve now better detailed and described this process within the section “Trajectory modelling and medication adherence estimation” (totally revised).

**Comment 5. It is not clear for me, why patient with only one OAD prescription had to be excluded. Even this is explained, I don’t understand the argument. (lines 106-108).**

**Response:** We understand the question and probably we explained the process of treating patients with a single prescription in an unclear manner, in the first version of the manuscript. To be clearer, we did not count patients with only 1 OAD prescription just in the persistence and trajectory analyses. This is because persistence is a time-to-event construct that requires at least two prescriptions to observe continued exposure and to define whether/when a permissible gap is exceeded. With a single prescription we cannot distinguish among: primary non-initiation, immediate discontinuation after the first script, very long coverage, or unobserved switching outside the index OAD. All these factors would misclassify persistence if we imposed an assumption. The same issue affects implementation trajectories (CMA9 in monthly windows). Indeed, A single prescription provides insufficient longitudinal information to form a stable trajectory and would influence the grouping. But it’s important to clarify that, these patients were not totally excluded from the study. These patients were counted under initiation (reported in the Results and SI) and described at baseline but not counted only from persistence and trajectory modelling to avoid differential misclassification. They represent a small fraction of the cohort (0.9%), so this decision does not materially affect the main estimates.

We understand that this justification was not well argued in the previous version of the manuscript, so we have detailed these specifications in the current revision of the manuscript.

**Reviewer #2 Comments**

**Comment 1: Although the research question addressed in the manuscript is important, the reporting of the approach is confusing, and there are multiple inconsistencies between the methods and results. My detailed concerns are outlined below
Response:** Dear Reviewer, thank you for this careful assessment. We appreciate the detailed comments provided in your review. Hence, considering your feedback, we have carried out an overall revision of the manuscript. Mainly, we clarified the analytical approach used step-by-step (data source, cohort construction, adherence definitions across initiation/implementation/discontinuation, CMA9 computation in monthly windows, trajectory modelling and selection criteria, and regression modelling). We have also aligned the methods and results so that each estimate reported now corresponds in a clear way. We standardized all the terminology (such as the index date, the study period and follow-up etc). Tables have been revised for readability, as per tables and figures’ footnotes (we included clearer descriptions). We also expanded the Supplementary Information with additional methodological detail (trajectory diagnostics and selection rationale, definitions of covariates, handling of missing data, and the exact clinical-window alignment for pre/post outcomes). Finally, we performed a full language edit to shorten long sentences. We hope these changes directly address your concerns about confusing reporting and any inconsistencies between methods and results. We are thankful for the improvements that your comments provided.

**Comment 2: Study Design. The design of the study is not clearly described. Baseline definition: The definition of “baseline” is incorrect. Lines 120–122: Profiles of subjects within each adherence trajectory were examined through an analysis of their baseline characteristics, including gender and age, their drug utilization patterns, and the addition of insulin therapy. With the index defined as initiation of OAD, what is meant by “addition of insulin therapy”? If patients were on insulin before OAD initiation, they should have been excluded. If insulin was added after OAD initiation, then this addition occurred outside of the baseline period.
Response:** Thank you for pointing this out. We agree and recognize this mistake. Indeed, in the first submission we added the number of patients starting insulin and time at insulin addition start improperly in Table 1. While, Table 1 (and also Supplementary Table S1) have to report baseline characteristics only of the overall OAD cohort also stratified by adherence groups.

We now amended these corrections. Specifically, in the revised manuscript we have restricted Table 1 to true baseline variables (sex, age, comorbidity, concomitant medicines and diagnosis, etc), and removed the insulin-start variable. Additionally, we now clarified in the manuscript that baseline values refer to the assessment window within 90 days before the index date (first OAD prescription). Also, we added the clarification on how we evaluated insulin addition. Namely, it was identified as any insulin prescription initiated after the index date (date of first OAD prescription) within the 12-month follow-up. We also make explicit in methods that the overall T2D cohort excluded patients with insulin before OAD initiation. Hence, insulin prescription was evaluated only after index as a follow-up characteristic and included in the regression as a post-index indicator, not as a baseline attribute. We also added a separate table to clarify this in the Supplementary material (new Table S4). You can find all the corresponding clarifications, additions and modifications in this new version of the manuscript and supplementary file.

**Comment 3: After carefully reading the manuscript, tables, and the responses to reviewer comments, it seems the design is actually cross-sectional for assessing the association between adherence and outcomes. This should be clearly stated.
Response:** We understand why parts of the first version could give that impression. However, the study is a retrospective longitudinal cohort, not cross-sectional. In this study we selected patients at the index date (date of the first OAD prescription) and followed each patient for 12 months. Hence, adherence was modelled longitudinally, using monthly CMA9 windows over the full follow-up to build the trajectory groups (implementation). Persistence/discontinuation is derived from the same time-stamped prescription series with a permissible-gap rule. Also, baseline values were assessed in the 90 days before index date, and values at the end of follow-up were collected in the 90 days after month 12. Although this, we recognise that descriptive summaries of values at the end of follow-up by trajectory may read as “snapshots” if timing is not explicit. We have therefore revised the manuscript to make the longitudinal design and time windows clearer. We also have now clarified that these values are descriptive longitudinal summaries.

We additionally now recognised that, as adherence trajectories and outcomes are constructed over the same 12-month horizon, we avoid causal language and interpret differences as associations. We corrected the whole manuscript as appropriate. All these clarifications now appear in the methods and in the limitations of the revised manuscript.

**Comment 4: Adherence Measures. The definitions and implementation of adherence measures are confusing. A few examples:
Lines 160–161: “Prescription histories for each single OAD medication over an observation period of 365 days (1 year) were calculated.”
Line 202: “In the persistence phase, 84.6% of the overall T2D cohort remained on therapy at the end of the one-year follow-up.”
It is unclear whether prescription history was captured across the full one-year period or only within the persistence phase.**
**Response:** In the revised manuscript we now clarified this. We stated explicitly that the prescription history was constructed for every patient across the entire 12-month follow-up from index date regardless of the adherence phase. From that single patient-level 12-month series we derived implementation trajectories and persistence/discontinuation assessment. We assessed this latter on the same 12-month series using a permissible-gap rule to determine whether the patient remained on therapy to month 12 (non-discontinuers) or discontinued earlier. Hence, the sentence “84.6% remained on therapy at the end of follow-up” refers precisely to this persistence estimate at 12 months.

We have now clarified this in the methods and results sections.

**Comment 5: In response to Reviewer 2, Q7, you stated:
“The group-based trajectory approach was applied to daily adherence data, specifically the continuous multiple-interval measure of medication availability (CMA), over a 12-month follow-up period…”
However, the corresponding section in lines 112–188 does not reflect this explanation. I did not realize adherence was measured monthly until I reached this response. The manuscript itself should make this point clear.
Response:** Yes, out earlier response was imprecise. In the study, CMA9 was computed in fixed, non-overlapping 30-day windows (monthly windows) across the 12-month follow-up, not daily. Also, K-means for longitudinal data to compute trajectories was applied to the 12 monthly CMA9 points per patient (months 1–12). We have clarified this throughout the whole manuscript, mainly in the methods section.

Additionally, we also clarified this in the footnote of the Figure S3/S4 legends.

**Comment 6: It is also unclear whether persistence and adherence were measured at the patient level or the drug level. Patient-level measurement is appropriate; however, the current description suggests drug-level measurement. If this is the case, how was switching handled? Were patients switching from one OAD to another classified as nonadherent?
Response:** In this study, each patient contributes exactly one adherence trajectory, which is derived from the prescription history of the patient’s index OAD (the first OAD prescribed at the index date). Thus, trajectory assignment is at the patient level, but the exposure that defines the trajectory is the index-drug prescription series.

More specifically, if a patient switched from the index OAD to a different OAD and stops refilling the index drug, the switch is treated as discontinuation of the index OAD and persistence ends at the first permissible-gap breach for the index drug. This approach avoids increasing adherence by counting any OAD exposure when the reference drug is no longer used. On the other hand, if a patient adds another OAD while continuing to refill the index OAD, this is not classified as non-adherence. The trajectory and persistence continue to be computed from the index-OAD series.

Furthermore, dose changes of the index OAD are handled by the CMA9 computation using the medicine-specific posology rules. When the prescriber increases the dose, the subsequent prescription increases the days’ supply, which updates the monthly coverage.

We have also specified that aspects in the methodology.

**Comment 7: Linear Mixed-Effects Regression Model. The description of the mixed-effects model is insufficient. The methods section does not specify whether baseline characteristics (e.g., disease severity) were controlled. The results section lacks a table summarizing the regression results. The figure provided is not sufficient to determine whether the observed outcomes truly differ across groups once baseline covariates are appropriately adjusted.
Response:**

We understand that the original description of the mixed-effects model was not complete. We have now expanded and clarified the methods, specifically statistical analyses. This was also made for the results section, specifically for the figure 1. Additionally, we added two new tables into the supplementary material (now Table S5 and Table S6).

More in detail, in the methods we now specified how we carried out the model. Hence, a linear mixed-effects regression for repeated HbA1c, with time since index (measured in months), adherence group, and their interaction as fixed effects. Also, baseline HbA1c (measured in the 90-day pre-index window) was used as a covariate, and patient-level random intercepts and slopes for time. We also clarify scale choices, the covariance structure and estimation approach. Baseline HbA1c was included as the primary marker of disease severity at index date. As a check, we ran a sensitivity model adding age, sex, and high-risk T2D status at baseline; the group x time contrasts were materially unchanged (direction and statistical significance).

In the supplementary material we added two tables to complete the Figure 1.

Table S5 (full mixed-model coefficients with 95% CIs and p-values (intercepts, main effects, and group x time interactions) and Table S6 (predicted monthly slopes (mmol/mol per month) and approximate 12-month changes by group, with 95% CIs). Results section now also reflects these additions.

**Comment 8: Conclusion. Overall, while the manuscript addresses a significant and relevant research question, substantial revisions are needed to clarify the study design, strengthen the description and interpretation of adherence measures, and provide a more transparent and detailed account of the statistical analysis. These changes are essential for the work to be interpretable and credible.**

**Response:** Thank for this summary comment and review provided. We have thoroughly revised the manuscript mainly to clarify study design, adherence calculation and the statistical analyses. Methods and results are now aligned. Also. Figures, tables and captions have been corrected, and the supplementary material includes implementations.

**Reviewer #3 Comments**

**Comment 1: The retrospective design using prescription refill data does not allow inference on whether medication was actually consumed. This fundamental limitation undermines the central claims linking adherence trajectories with improved clinical outcomes.**

**Response:**

We agree that electronic healthcare records cannot prove medication ingestion. The data source used contains primary care prescribing records rather than pharmacy dispensing claims, and this is now stated and discussed explicitly in the methods and expand it in the limitations. Albeit this, the aim of this study is to characterize adherence trajectories from routine care data and examine their association with clinical parameters in a large, real-world primary-care population. This objective aligns with the EMERGE guideline and the ABC taxonomy (*references nr 5 and nr 6 in the manuscript*), where adherence measurement from longitudinal prescription histories (CMA/PDC/MPR frameworks) is recognised as a standard (especially in chronic conditions) (*references from 1 to 3 here below*).

Additionally, in T2D, antihyperglycaemic therapy is typically continuous and long-term. Within this context, repeated prescriptions over 12 months carry meaningful information about continued intended use. Of course, these prescriptions do not verify intake, but they provide an accepted proxy for availability/implementation of the therapy.

Hence, the consistent association observed in this study (higher adherence trajectories co-occurring with lower mean HbA1c and LDL over time) supports the validity of the adherence proxy in this setting and is in line with literature based on administrative data *(reference 4 here below*).

Also, as you suggested, to respect the inferential limits of prescribing data, we have now revised the wording throughout the manuscript to avoid any causal language. For example, statements that could be read as causal ( “impact” or “effect”, “improved”) have been replaced with statistically neutral phrasing that reflects associations estimated under our models ( “were associated with”, “had lower average levels”, “showed better control”).

*References:*

*1) Anghel LA, Farcas AM, Oprean RN. An overview of the common methods used to measure treatment adherence. Med Pharm Rep. 2019 Apr;92(2):117-122. doi: 10.15386/mpr-1201.*

*2) Pednekar PP, Ágh T, Malmenäs M, Raval AD, Bennett BM, Borah BJ, Hutchins DS, Manias E, Williams AF, Hiligsmann M, Turcu-Stiolica A, Zeber JE, Abrahamyan L, Bunz TJ, Peterson AM. Methods for Measuring Multiple Medication Adherence: A Systematic Review-Report of the ISPOR Medication Adherence and Persistence Special Interest Group. Value Health. 2019 Feb;22(2):139-156. doi: 10.1016/j.jval.2018.08.006.*

*3) Elseviers M, et al. (ed. II). Drug utilization research: methods and applications. John Wiley & Sons, 2024.*

*4) Pladevall M, Williams LK, Potts LA, Divine G, Xi H, Lafata JE. Clinical outcomes and adherence to medications measured by claims data in patients with diabetes. Diabetes Care. 2004 Dec;27(12):2800-5. doi: 10.2337/diacare.27.12.2800.*

**Comment 2: The definition of initiation as “at least two prescriptions” is arbitrary and excludes patients who discontinued legitimately after one prescription. This exclusion introduces bias and potentially misclassifies patients, weakening external validity.**

**Response:** We understand this point, and we recognise that probably we explained this process of treating patients with a single prescription in an unclear manner, in the first version of the manuscript.

First, to avoid any misunderstanding, we confirm that in our study initiation is not defined as ≥2 prescriptions. Initiation is defined as receipt of the first primary-care prescription for an OAD (at index date). Patients with only one OAD prescription during follow-up are therefore initiators and are reported in the initiation phase and described at baseline(see revised results and supporting information file). The requirement of ≥2 prescriptions apply only to persistence estimation and trajectory modelling of implementation, not to initiation. This is a methodological necessity. This is because persistence is a time-to-event construct that requires at least two prescriptions to observe continued exposure and to define whether/when a permissible gap is exceeded. With a single prescription we cannot distinguish among: primary non-initiation, immediate discontinuation after the first script, very long coverage, or unobserved switching outside the index OAD. All these factors would misclassify persistence if we imposed an assumption. The same issue affects implementation trajectories (CMA9 in monthly windows). Indeed, A single prescription provides insufficient longitudinal information to form a stable trajectory and would influence the grouping. But it’s important to clarify that, these patients were not totally excluded from the study. These patients were counted under initiation (reported in the Results and SI) and described at baseline but not counted only from persistence and trajectory modelling to avoid differential misclassification. They represent a small fraction of the cohort (0.9%), so this decision does not materially affect the main estimates.

We understand that this justification was not well argued in the previous version of the manuscript, so we have detailed these specifications in the current revision of the manuscript.

**Comment 3: The justification for selecting four adherence clusters is insufficient. Although indices (Calinski-Harabasz, Davies-Bouldin) were applied, the process appears exploratory and lacks robustness testing (e.g., bootstrapping, validation in external datasets). Without external replication, the stability of clusters and generalizability of identified trajectories are questionable.**

**Response:** We estimated group-based longitudinal k-means solutions for k = 2-6 (i.e. 2 to 6 groups) using 12-month CMA9 series (one value per month per patient). We compared solutions with a multi-criteria panel of internal indices (standardized Calinski–Harabasz CH- CH2- CH3, Ray-Turi, Davies-Bouldin) and clinical interpretability of centroid shapes. Also, we re-estimated the k=4 solution to assess stability with multiple random starts (n= 100) and bootstrap resampling (B= 200 resamples with replacement). This was done by using the Jaccard similarity of cluster membership as the primary stability metric (cut-offs: ≥0.75 stable, 0.60-0.75 moderate). We also performed a split-sample reproducibility check. Hence, centroids were derived in a random half-sample and the hold-out half was assigned by nearest centroid. Sensitivity analyses were performed in this way: 1) the monthly CMA9 construction (±3-day tolerance around month boundaries); 2) application of a light 3-point moving average (yes/no); 3) a minimum cluster size of 5% to avoid small clusters. Finally, the selection rule was the smallest k with jointly favourable indices, stable membership (Jaccard ≥ 0.75), and distinct (also clinically interpretable) trajectory shapes.

We now provided to add all these clarifications in the methods and results sections, and also within the footnotes of the S3 Figure in Supporting information.

**Comment 4: Key confounders such as socioeconomic status, educational attainment, and health literacy—well-established determinants of adherence—are absent. Relying solely on comorbidity indices (Charlson, multimorbidity count) does not sufficiently control for patient-related factors, leading to potentially spurious associations between “perfect adherence” and better outcomes.**

**Response:** We agree with this observation as adherence is multi-determinant and influenced by several factors related to therapy, condition, patient, health system, and socioeconomics. Also, patient-related factors such as socioeconomic status, education, and health literacy are surely important, as also addressed by the WHO’s report on adherence to long-term therapies. These variables are not captured in within the data source we used for this study as this info are not reported in the prescribing and clinical records. We adjusted the analyses for constructs that are available and clinically meaningful in routine care data, namely overall morbidity burden with the validated Charlson index, multimorbidity count, polypharmacy/concomitant medicines, high-risk T2D status per national guidance, age, and sex.

We now expanded the limitation section to declare explicitly unmeasured confounding factors.

**Comment 5: Clinical outcomes were restricted to HbA1c, LDL, blood pressure, and BMI. Other relevant T2D outcomes such as microvascular complications, hospitalizations, and hypoglycemia were ignored, narrowing the clinical relevance of the findings.**

**Response:** We agree that other outcomes, such as microvascular/macrovascular events, hospitalizations, hypoglycaemia are clinically important. Our aim, however, was to test whether adherence trajectories are associated with guideline-endorsed intermediate outcomes(mainly HbA1c, LDL, BP, and BMI as reported from the Dutch national guidelines NHG). This is because these parameters should routinely monitored in Dutch primary care as recommended by NHG for ongoing T2D management. These parameters are also validated surrogates that predict future micro- and macrovascular risk and hospitalisation risk. Of course, we recognise the importance of micro/macrovascular events and re-hospitalizations

Reason why we already have examined these in our prior study using different data source. Please refer to this ref below:

*Guerrero-Fernández de Alba I, Orlando V, Monetti VM, Mucherino S, Gimeno-Miguel A, Vaccaro O, Forjaz MJ, Poblador Plou B, Prados-Torres A, Riccardi G, Menditto E. Comorbidity in an Older Population with Type-2 Diabetes Mellitus: Identification of the Characteristics and Healthcare Utilization of High-Cost Patients. Front Pharmacol. 2020 Nov 30;11:586187. doi: 10.3389/fphar.2020.586187.)*

**Comment 6: HbA1c reflects glycaemic control over 2–3 months, yet persistence and implementation measures were calculated annually. This temporal discordance weakens causal interpretations.**

**Response:** We understand this important observation. We have now added some explicit clarifications in methods section.

Hence, we agree that HbA1c integrates glycaemic control over about 8-12 weeks and therefore requires careful temporal alignment with exposure. We would like to give two clarifications.

Firstly, although the observation horizon was 1 year, implementation was calculated as CMA9 in consecutive monthly windows and then used to derive longitudinal trajectories (we have now better addressed this aspect in the methodology). Therefore, exposure varies over the course of the year and is not a single annual average.

Secondly, our HbA1c analysis used a linear mixed-effects model that incorporated all available HbA1c timestamps during follow-up, with random intercepts (and slopes) at the patient level and the adherence trajectory group as a fixed effect. This related contemporary HbA1c trends to the time-varying implementation pattern over the same 12-month span. For pre/post descriptive summaries, we also aligned measurements to clinical windows: the HbA1c closest to baseline (within 3 months before) and the HbA1c closest to the end of follow-up (within 3 months after). In the revised manuscript, we made this alignment explicit and moderated causal language, defining the results as associations.

You can now find all these adjustments in this revised version of the manuscript.

We also added this clarification in the limitation section.

**Comment 7: The authors claim that trajectory modelling is novel, yet several recent studies have applied similar approaches in T2D and other chronic diseases. The manuscript does not convincingly demonstrate methodological innovation or clinical impact beyond confirming established knowledge.**

**Response:** We recognise that group-based longitudinal trajectory methods are well established in the adherence literature so far. Hence, we acknowledged and cited multiple prior applications, both within T2D and also across other chronic cardiometabolic conditions (eg, atrial fibrillation/DOACs, coronary disease, heart failure, statins [references 12, 36, 53, 54]). Indeed, our intent was not to claim methodological novelty per se, but original contribution is in how we implement and link trajectories in this setting.

Specifically, we used CMA in the new version 9 (instead of the widely PDC/MPR indices) to construct monthly, carryover-aware adherence series. Also, aligning with EMERGE/ABC guidelines, we assessed adherence within all adherence phases using also trajectory application. Also, we related the resulting trajectories to multiple guideline clinical markers simultaneously (HbA1c, LDL, BP, BMI) using all time-stamped measurements over 12 months.

We have revised the manuscript to avoid implying method novelty di per se and to present the work as an incremental advance combining an updated adherence metric with broader clinical evaluation across the full adherence process.
