# Supplementary material for: Medication adherence trajectories and association with risk factors and clinical outcomes in type 2 diabetes treatment
Source: PLoS One. 2026 Feb 20;21(2):e0342056. doi: 10.1371/journal.pone.0342056 (PMC12923057; doi:10.1371/journal.pone.0342056)
Supplement: Attachment — Submitted filename: Response_to_Reviewers_auresp_2.docx [file pone.0342056.s017.docx]

**Response to Reviewers**

**PONE-D-25-41866**

**Medication adherence trajectories to predict risk factors and clinical outcomes in type 2 diabetes treatment**

**Academic Editor Comments**

**Please read the reviewers carefully and resubmit if you are able to address them.**

**Response:** Dear Academic Editor, we appreciate this response and we’re certainly able to address these additional requests from the reviewer #1. We’ve made all the pertaining modifications and improvements. We prepared a revised version of the manuscript. Below in this report you’ll find all the detailed responses to reviewers also addressed in the manuscript.

**Journal Requirements**

**Comment 1. If the reviewer comments include a recommendation to cite specific previously published works, please review and evaluate these publications to determine whether they are relevant and should be cited. There is no requirement to cite these works unless the editor has indicated otherwise.**

**Response:** Dear editors, thank you for your advice. We have not received any suggestions from reviewers regarding the addition of references.

**Comment 2. Please review your reference list to ensure that it is complete and correct. If you have cited papers that have been retracted, please include the rationale for doing so in the manuscript text, or remove these references and replace them with relevant current references. Any changes to the reference list should be mentioned in the rebuttal letter that accompanies your revised manuscript. If you need to cite a retracted article, indicate the article’s retracted status in the References list and also include a citation and full reference for the retraction notice.**

**Response:** Dear editors, thanks for the notification. We confirm that we have checked the reference list as suggested and have not made any changes.

**Reviewers’ Comments**

**Reviewer #1 Comments**

**Comment 1. The text of Abstract has improved and now clearly structured. The definition of follow-up is now clarified in methods section.**

**Response:** Dear Reviewer, many thanks for your positive feedback. In this revised submission, we have also addressed the additional points raised in your second round of comments, with corresponding changes integrated.

**Comment 2. The system of prescription differs in different countries. Therefore, the prescription system in Netherlands should be clarified in methods section. For how long period is one prescription? For example, in my country prescription of insulin is for one year and for oral medication it is two years. After that the physicians have to renew the prescription of each medication. Please clarify the detailed prescription system in Netherlands.**

**Response:** Dear Reviewer, we understand this specification. We added this concept in the method section. Specifically, in Dutch primary care, chronic therapies are issued as repeat prescriptions (“herhaalrecept”). In standard practice, a repeat authorization typically covers up to 12 months. Community pharmacies, however, dispense the medications approximately 1-3 months per prescription, based on the insurer's policy and clinical opinion. Insulins and oral antidiabetic drugs follow the same framework. The prescription authorisation may cover a year, but the dispensed quantity per issue is generally limited to ~3 months. These explain why prescription dates recur periodically in the electronic record and support our construction of coverage periods from successive prescription records. We therefore computed days of supply from the prescribed regimen and dates, allowing carry-over, and assessed persistence via a permissible-gap rule over the 12-month follow-up. We have now specified this in the methods clarifying these points.

**Comment 3. The authors have edited the text, but some of the paragraphs are still complex. The authors could further condense sentences to make it easier to read.**

**Response:** Dear Reviewer, thanks for this remark. We conducted an additional full read-through of the main text and further simplified several long or complex sentences to improve readability.
