# Supplementary material for: Medication adherence trajectories and association with risk factors and clinical outcomes in type 2 diabetes treatment
Source: PLoS One. 2026 Feb 20;21(2):e0342056. doi: 10.1371/journal.pone.0342056 (PMC12923057; doi:10.1371/journal.pone.0342056)
Supplement: Attachment — Submitted filename: Response_to_Reviewers_auresp_3.docx [file pone.0342056.s018.docx]

**Response to Reviewers**

**PONE-D-25-41866**

**Medication adherence trajectories to predict risk factors and clinical outcomes in type 2 diabetes treatment**

**Academic Editor Comments**

**Comment 1. We only managed to get 1 reviewer who recommended major revision
Please assess and resubmit if you are able to address the issues raised.**

**Response:** Dear Academic Editor, we’ve made all the pertaining modifications and improvements. We prepared a revised version of the manuscript. Below in this report you’ll find all the detailed responses to reviewers also addressed in the manuscript.

**Journal Requirements**

**Comment 1. If the reviewer comments include a recommendation to cite specific previously published works, please review and evaluate these publications to determine whether they are relevant and should be cited. There is no requirement to cite these works unless the editor has indicated otherwise.**

**Response:** Dear editors, thank you for your advice. We have not received any suggestions from reviewers regarding the addition of references.

**Reviewers’ Comments**

**Reviewer #4 Comments**

**General comment: This is my first review of this paper assessing medication adherence trajectories and their association with characteristics and clinical outcomes in type 2 diabetes. The writing is generally clear, and the objectives and methods are reasonably sound. I have several major and minor comments to improve clarity and relevance of the manuscript.**

**Response:** Dear Reviewer, we greatly appreciate both your general and specific comments on the manuscript. We have made the requested changes to improve the clarity of the manuscript. We hope you will appreciate the revised version of the resubmitted manuscript. Below you will find point-by-point responses to each of your comments. Thank you.

**Comment 1. Title: The current title “Medication adherence trajectories to predict risk factors and clinical outcomes in type 2 diabetes treatment.” is misleading because the trajectories are assessed concurrently with clinical outcomes. Therefore, the authors cannot claim that adherence trajectories predict outcomes; they can only state that associations exist. Additionally, the manuscript uses the words correlation and association interchangeably. The authors should be more precise about the type of analysis performed, the specific resulting measures and they should interpret them correctly.**

**Response:** Dear Reviewer, thanks to note this important point. We agree that our study assesses concurrent associations rather than prediction or causal effects. We have therefore: i) revised the title to remove “predict” and ii) standardised wording across the manuscript to use “association/associated with” and avoid “predict/impact”. Also we now used “association” rather than “correlation” (except when referring to correlation as a statistical quantity). The manuscript now explicitly states the observational nature of the design and that we refrain from causal claims. These changes are now reflected in the title and within the whole manuscript.

**Comment 2. The previous reviewer asked clarifications on how the prescription system works in the Netherlands, particularly regarding prescription duration. The authors explained that “therapies are issued as repeat prescriptions that typically covers up to 12 months and that community pharmacies, dispense these medications approximately 1-3 months per prescription ». However, it remains unclear what type of data are available in the Nivel Primary Care Database (Nivel-PCD): prescription data (what the doctor prescribed), dispensation data (what the pharmacist provided) or both? The authors should use precise terminology (e.g., medication supply, medication claim, medication dispensed, medication prescribed) throughout the manuscript to avoid confusion.**

**Response:** Dear Reviewer, we agree that a coherent terminology is essential for the clarity of the manuscript. Please, consider that the Nivel Primary Care Database (Nivel-PCD) contains general practice electronic health record data, including prescribed medications recorded by GPs (date, drug/ATC, dose instructions, and quantity/duration). Hence, Nivel-PCD does not contain pharmacy dispensing claims. We have now standardised wording throughout the manuscript to be sure to avoid terms implying dispensing (such us medication refill etc). Moreover, days of supply and coverage were derived from the GP prescription record (prescribed daily dose and recorded duration/quantity), and adherence was computed on that basis. Persistence was assessed on the same prescription series using a permissible-gap rule, and switching to a different OAD with cessation of index prescriptions was treated as discontinuation of the index OAD. We have also now clarified how repeat prescriptions work in Dutch primary care. Generally, GPs authorise repeat therapy (typically for up to 12 months), while community pharmacies dispense in shorter amounts (often 1–3 months per issue). Finally, the database we used for the study captured the prescription authorisations and dates from GPs, not the pharmacy dispenses. All of these clarifications have been now added to the method section.

**Comment 3. Line 79: Are you certain that 90% of 1.1 million patients in 2019 had type 2 diabetes? Or do you mean that among patients with diabetes, 90% had type 2? This needs clarification.**

**Response:** We understand that the sentence may have caused some confusion. Referring to reference no. 19 of CBS statistics Netherlands, we confirm that the percentage referred to the total number of diabetic patients. We have therefore reworded the sentence more precisely as follows: “In the Netherlands, type 2 diabetes accounts for 82% of all diabetes cases [19], with negative consequences for public health [20–22]”.

**Comment 4. Line 81-84: It is unclear whether you are referring to the Nivel Primary Care Database or your study population when discussing inclusion criteria.**

**Response:** Yes definitely. We referred to our data source, which is Nivel-PCD. We have now specified the data source more clearly in the inclusion criteria, where suggested. Thanks.

**Comment 5. Line 107-108: Why was the initiation phase defined as at least two prescriptions within one year? Initiation typically refers to when the patient takes the first dose of the prescribed medication. To assess initiation accurately, both prescription and dispensation data are needed. Otherwise, using the term “initiation” is misleading. This should be clarified.**

**Response:** We understand the reason you are raising this point. According the EMERGE guidelines, *initiation* is the patient’s first dose and that confirming it would require dispensing (or intake) information. Albeit this, our dataset contains prescription records issued by GPs, not pharmacy dispensing claims. That said, as no dispensing data was available, we used a proxy: having a second prescription from GPs presumably means that the first dose was dispensed and taken. We now better declared in the study methods that “Initiation phase was calculated as the occurrence of at least two prescriptions of the index oral antidiabetic drug (OAD) within the one-year follow-up period, since dispensing data were not available” to avoid a misleading use of the term. We have also now better specified within the study limitation section that “The analyses were based on a data source which contains prescription records of the whole Dutch population. However, prescription records cannot confirm whether patients took or not their prescribed medication. To address this, we considered at least two prescriptions within the observation period as a proxy for treatment initiation and continuation. Hence, days of supply were built on OADs-prescriptions.”.

**Comment 6. Lines 116-117: The authors state “Patients with only one prescription during follow-up were reported under initiation but excluded from persistence and implementation estimations.” While it is understandable why implementation was not calculated for these patients, why was persistence not assessed? These patients clearly did not persist with their treatment beyond the initial dispensation period.**

**Response:** As according to the EMERGE guidelines, persistence is a time-to-event, hence, in this study, we measured it based on a permissible-gap rule, which requires at least two prescriptions to identify when a gap first exceeds 2× the expected coverage. With only one prescription, we cannot observe a discontinuation date without imputing it from a single record (which risks misclassification given heterogeneity in days-supply and possible unobserved switches). For this reason, patients with one prescription were reported under treatment start evaluation (based on prescribing data) but excluded from persistence and trajectory estimation. They represent about 0.9% of the cohort, so the impact on overall estimates is minimal. We have now made this rationale more explicit in methods.

**Comment 7. Lines 124-126: The cluster analysis is poorly explained and should be clarified.**

**Response:** Dear reviewer, we’ve now more in depth explained the cluster analysis approach where indicated. More details are also described within the statistical analysis. We added these paragraph as follows: “Clustering method used consisted of a set of multivariate techniques aimed at recognising and grouping homogeneous items in a data set by using non-parametric statistics [10, 24–26]. The clustering approach grouped patients with similar month-to-month adherence rates over the first year of the OAD-therapy. Each cluster was summarized by a centroid, i.e., the typical adherence curve formed by averaging the profiles of its members. Then, the clusters were labelled by the shape of their centroid to reflect recognizable behaviours in adherence. Hence, cluster analysis for adherence implementation measurement allowed the identification of groups of T2D patients with common characteristics (e.g., potential determinants or predictors of risk) [14, 24–26]. This approach linked heterogeneous individual adherence patterns into a small set of clinically interpretable behaviours.”.

**Comment 8. Lines 162-163: This limitation - that adherence and outcomes were measured over the same period and that outcomes may have occurred before exposure - should be explicitly stated in the limitations section.**

**Response:** Yes, we understand that there is the possibility that some outcome measurements may occur before subsequent adherence changes. We’ve now more explicitly stated this in the limitation section.

**Comment 9. Line 178: Not all sulfonylureas are taken once daily. This should be noted as a limitation, given that the database lacks information on directions for use or medication days’ supply.**

**Response:** Thank you for this helpful point. We agree that dosing within classes (such as sulfonylureas) varies by molecule and formulation. We have to specify that in our dataset, days of supply were primarily derived from prescription-level fields: namely the recorded prescription duration (days) and the prescribed daily dose. We of course checked that the posology was in accordance with the national guidelines. Also, when these fields were missing, we infer coverage using molecule/formulation-specific dosing from national guidance and product information, together with the timing of successive prescriptions. For examples, for sulfonylureas, modified-release products were treated as once-daily, whereas immediate-release formulations were treated as once- to twice-daily. This was consistent with product characteristics. We understand that this approach must be better specified within the manuscript, hence, we have now revised the methods section to reflect this and also added limitation noting potential of coverage.

**Comment 10. Lines 191-194: This section is difficult to understand, even for readers familiar with this methodology. It should be rewritten to improve clarity.**

**Response:** We’ve totally rewritten the paragraph as follows: “Cluster stability was assessed in three steps. First, k-means was run with 100 random initialisations to reduce dependence on starting values. Second, bootstrap resampling was applied (200 samples with replacement). For each bootstrap sample, the solution was compared with the full-sample solution using the Jaccard index for cluster membership. Specifically, values ≥0.75 were considered stable and values 0.60–0.75 moderately stable. Third, a split-sample check was performed: centroids were estimated in a random 50% training subset, and the remaining 50% were assigned to the nearest centroid. Finally, agreement with the full-sample assignment was then evaluated.”

**Comment 11. Line 210: State that p-values<0.05 were “considered” statistically significant.**

**Response:** We added this clarification. Thanks.

**Comment 12. Lines 227-228: Again, this does not represent initiation per se. This should be revised.**

**Response:** We addressed this point in response to the earlier comment on the definition of initiation. We have now clarified in the methods that initiation in this study refers to treatment start based on prescribing data, not confirmed medication intake, and that this definition aligns with what can be observed in prescription records. We also acknowledged this better within the limitation section.

**Comment 13. Line 233: FU (means follow-up)? This term should be written in full for clarity.**

**Response:** Yes, we have now corrected it and written it out in full.

**Comment 14. Lines 244-245: This sentence contains two instances of “after”. One should be removed.**

**Response:** We’ve amended it. Thanks.

**Comment 15. Lines 281-290: The authors report LDL, BMI and blood pressure control at the beginning and the end of the 12-month follow-up period. This seems questionable. What is the rationale for linking adherence to OAD with LDL, BMI and blood pressure control? The authors claim they are the only ones to have perform that type of analysis, which they say is a strength of their work (lines 373-377). But what is the novelty/strength if it does not make sense? This is a major flaw of this paper, certainly not a strength.**

**Response:** We agree that HbA1c is the primary glycaemic outcome for OAD adherence, and we do not claim that OAD adherence causes changes in LDL, blood pressure or BMI. The aim of this study was different: in routine T2D care, cardiometabolic risk-factor control (HbA1c, LDL, BP, weight) is monitored together and managed multifactorially. Many patients in our study cohort received concomitant lipid-lowering and antihypertensive therapies, and adherence to OADs often coexists with broader treatment adherence and care engagement. Also for this reason, we treated LDL, BP and BMI as secondary, descriptive outcomes, used to profile clinical risk-factor control across adherence groups (not to infer drug-specific effect).

To avoid any implication of causality, we have now revised the manuscript in tha way: i) position LDL, BP and BMI explicitly as exploratory secondary outcomes. ii) Make clear that analyses are associational and descriptive. iii) Acknowledge that changes in these outcomes may reflect multiple concurrent influences (lifestyle, intensification of non-glycaemic medications, clinical follow-up), beyond OAD adherence.

All these addition/modifications were made within the introduction, methods, results and limitation section.

**Comment 16. Lines 295-305: Results in this section are reported with three decimal places, whereas the rest of the manuscript uses two. This should be standardized.**

**Response:** We’ve now amended it. We changed everything to two decimal places to be consistent throughout the manuscript. Thanks.

**Comment 17. Lines 313-318: These sentences and their connections are unclear and should be revised for better flow.**

**Response:** Dear reviewer, we understand this clarification request. We’ve now changed the paragraph as follows: “This study mapped medication adherence across its three phases (initiation, implementation, and persistence/discontinuation) during the first year of treatment in newly diagnosed T2D receiving OADs. The three adherence phases were evaluated in accordance with the most recent adherence guidelines (the EMERGE guidelines [5] and the ABC taxonomy [6]). Using longitudinal trajectories, four common adherence behaviours were identified (perfect adherence, slow decline in adherence, low adherence, slow increase in adherence). Higher-adherence patterns were associated with better risk-factor control over time, particularly lower HbA1c and LDL cholesterol. The OAD-therapy initiation was high (99%), and one-year persistence was consistent with prior T2D reports (70–80%) when considered across adherence subgroups, with marked variation between behaviours [32–34].”

**Comment 18. Line 421-423: The authors state that their “findings confirm that high adherence in patients with type 2 diabetes is associated with better clinical outcomes…”. Was a study needed to confirm this? What new insights does this study provide and how can they be applied?**

**Response:** We know that the association between higher adherence and better glycaemic control is well established and documented in the literature so far, and we do not claim novelty on that point. Our added value is elsewhere. First, we mapped adherence across all three EMERGE/ABC phases using CMA9 in monthly windows. Second, we identified four longitudinal implementation patterns in a real-world, newly diagnosed T2D cohort and quantified their very different persistence profiles. Third, we linked these patterns to HbA1c trajectories using a mixed-effects model that adjusts for baseline HbA1c, showing distinct slopes over time. And also described LDL, BP and BMI as secondary, routine risk-factor indicators to give clinical context. In practice, these trajectory patterns can help clinicians prioritise patients for adherence support and tailor follow-up, and they can inform population monitoring in primary care. We stressed this in the conclusion section.

**Comment 19. References 41 and 46 are duplicates and should be corrected.**

**Response:** Thank you for bringing this to our attention. We have deleted the duplicate.
