# Supplementary material for: Medication adherence trajectories and association with risk factors and clinical outcomes in type 2 diabetes treatment
Source: PLoS One. 2026 Feb 20;21(2):e0342056. doi: 10.1371/journal.pone.0342056 (PMC12923057; doi:10.1371/journal.pone.0342056)
Supplement: Attachment — Submitted filename: Response_to_Reviewers_auresp_4.docx [file pone.0342056.s019.docx]

**Response to Reviewers**

**PONE-D-25-41866**

**Medication adherence trajectories and association with risk factors and clinical outcomes in type 2 diabetes treatment**

**Academic Editor Comments**

**Comment 1. We have invited multiple reviewers and 2 of them have came back with some comments. Please address them and resubmit if applicable.**

**Response:** Dear Academic Editor, we’ve made all the pertaining modifications and improvements. We prepared a revised version of the manuscript. Below in this report you’ll find all the detailed responses to reviewers also addressed in the manuscript.

**Journal Requirements**

**Comment 1. If the reviewer comments include a recommendation to cite specific previously published works, please review and evaluate these publications to determine whether they are relevant and should be cited. There is no requirement to cite these works unless the editor has indicated otherwise. 
Please review your reference list to ensure that it is complete and correct. If you have cited papers that have been retracted, please include the rationale for doing so in the manuscript text, or remove these references and replace them with relevant current references. Any changes to the reference list should be mentioned in the rebuttal letter that accompanies your revised manuscript. If you need to cite a retracted article, indicate the article’s retracted status in the References list and also include a citation and full reference for the retraction notice..**

**Response:** Dear editors, thank you for your advice. We have not received any suggestions from reviewers regarding the addition of references. We also checked all the reference list. Many thanks.

**Reviewers’ Comments**

**Reviewer #1 Comments**

**General comment: I commented previously that the prescription system in Netherland should be clarified in methods section. Still, it is not clear for me. Because The prescription of medication and its practices are the foundation of the entire article, this should be described in sufficient detail in the methods section. Otherwise, I am now satisfied with the revised text.**

**Response:** Dear Reviewer, we understand your point. We have added further details on these aspects in the methods section, in the subsection Data source and study population selection, as follows: “This study was carried out based on data sourced from Nivel Primary Care Database (Nivel-PCD). Nivel-PCD systematically collects data from approximately 500 general practices in the Netherlands (around 10% of the Dutch population) including prescribed medications recorded by general practitioners (GPs) [18]. Hence, Nivel-PCD contains GPs’ prescribing records, namely medications ordered by the GPs within the Dutch health system. Pharmacy dispensing/claims data is not captured in Nivel-PCD. In Dutch primary care, long-term therapies are issued as repeat prescriptions with an authorisation typically valid for up to 12 months. Community pharmacies generally dispense 1-3 per month quantities per issue. Insulins and oral antidiabetic drugs (OADs) follow the same framework. Each prescription record in Nivel-PCD includes the calendar date, Anatomical Therapeutic Chemical Classification (ATC) code, prescribed daily dose/instructions, and intended duration or quantity. These fields were used to derive days of coverage per prescription and to build 12-month prescription histories from the index date (first OAD prescription). For this study, these data were integrated with patient characteristics, healthcare consultations, patient morbidity profiles, laboratory results, and the primary or secondary healthcare provider managing diabetes care.” To be more clear we also added the recent reference regarding the prescription info available within the Nivel-PCD in the Netherlands (now reference no 18): Vanhommerig JW, Verheij RA, Hek K, Ramerman L, Hooiveld M, Veldhuijzen NJ, Veldkamp R, van Dronkelaar C, Stelma FF, Knottnerus BJ, Meijer WM, Hasselaar J, Overbeek LI. Data Resource Profile: Nivel Primary Care Database (Nivel-PCD), The Netherlands. Int J Epidemiol. 2025 Feb 16;54(2):dyaf017. doi: 10.1093/ije/dyaf017.

**Reviewer #4 Comments**

**General comment: The authors have adequately addressed most of my comments. Here are some minor revisions that still need to be done.**
**Response:** Dear Reviewer, again, we greatly appreciate these additional specific comments on the manuscript. They’re precise and pertinent and helped to improve the clarity of the manuscript. We made all requested changes and hope you will appreciate the revised version of the resubmitted manuscript. Below you will find point-by-point responses to each of your comments. Thank you.

**Comment 1: Line 81: The term ATC was added here, but is defined only on line 91. Please move the definition to the first occurrence.**
**Response 1:** Thanks. We’ve now cited in full the term ATC in the first occurrence.

**Comment 2: Line 113: What was the initiation date?**

**Response 2:** The initiation date is the index date, which is the calendar date of the patient’s first prescription of the index OAD. This date marks the start of follow-up. As the dispensing data were unavailable, evidence of the initiation phase was operationalised as having ≥2 prescriptions within 12 months after the index date; this does not change the initiation date itself. If the patient had more than 2 following OAD prescriptions we flagged the patient as “initiator”, hence the index date corresponds to the OAD therapy initiation for this patient. We explained this in the manuscript were suggested.

**Comment 3:**  **Lines 128-132: The definition and calculation of implementation should be moved here, not into the statistical analysis section.
Response 3:** We agree with this perspective. We have in fact moved the entire description and definition of the implementation phase from statistical analysis to the paragraph entitled “Trajectory modelling and medication adherence estimation”. Thank you.

**Comment 4: Lines 171, 298, 299, 300, 317, 338: FU should be written in long.**

**Response 4:** We have now always written the word "follow-up" in full.

**Comment 5: Statistical analysis (see my comment above): Much of what is written in this section should be moved earlier, when these variables are defined, notably lines 179-186 and 188-191.**

**Response 5:** As said, we agree. We’ve moved majority of these descriptions to the statistical analysis section.

**Comment 6: Line 207: times a day**

**Response 6:** Amended. Thanks.

**Comment 7:** **Line 266: (98.8%) at the end of...**
**Response 7:** Amended. Thanks.

**Comment 8:** **Table 2: Nr is not defined under the table and should probably be abbreviated by no. or N**

**Response 8:** We changed it with “N”. Thanks.

**Comment 9: Lines 330-332: Logistic regression models and OR are not mentioned and defined in the analysis section. This information should be presented before.**
**Response 9:** We added a paragraph with the pertinent specifications within the statistical analysis section, as follows: “Multivariate logistic regression was performed to identify factors associated with adherence clusters. A stepwise selection procedure was used to select covariates to be included in the models. These covariates included: sex, age category (<25, 26–39, 40–59, 60–79, ≥80 years), high-risk T2D status per NHG guidance [27], hypertension at baseline, baseline HbA1c within target (≤53 mmol/mol; 7%), baseline blood pressure within target (<140/90 mmHg), and insulin addition during follow-up (post-index indicator). Results are presented as adjusted odds ratios (aORs) with 95% confidence intervals and two-sided p-values (α=0.05).”

**Comment 10:** **Line 332: There are still three decimal places there, and elsewhere as well.
Response 10:** We have corrected and double-checked the entire paper, including the supplementary material. Thanks.

**Comment 11:** **Table 3: aOR and 95% CI are not defined under the table, and there is a 0.477 (three decimals).**

**Response 11:** We’ve now defined it at the bottom of the table and amended the numbers with 3 decimals. Thanks.

**Comment 12: Line 344: p<0.005 should probably be p<0.05**

**Response 12:** Yes, amended.

**Comment 13: Lines 363-368: The section repeats what was added in lines 350-352.**
**Response 13:** We merged these two paragraphs in one. Thanks.

**Comment 14: Line 374: "better" or "greatest" should probably be removed.
Response 14:** We’ve now reworded.

**Comment 15: Line 473: diagnosed with type 2 diabetes?**

**Response 15:** Yes, amended.
